# Protection of Photosynthesis by Halotolerant *Staphylococcus sciuri* ET101 in Tomato (*Lycoperiscon esculentum*) and Rice (*Oryza sativa*) Plants During Salinity Stress: Possible Interplay Between Carboxylation and Oxygenation in Stress Mitigation

**DOI:** 10.3389/fmicb.2020.547750

**Published:** 2021-01-08

**Authors:** Zarin Taj, Dinakar Challabathula

**Affiliations:** Department of Life Sciences, School of Life Sciences, Central University of Tamil Nadu, Thiruvarur, India

**Keywords:** plant growth-promoting bacteria, salinity stress, *Oryza sativa*, *Lycoperiscon esculentum*, photosynthesis, photorespiration

## Abstract

Tomato (*Lycoperiscon esculentum*) and rice (*Oryza sativa*) are the two most important agricultural crops whose productivity is severely impacted by salinity stress. Soil salinity causes an irreversible damage to the photosynthetic apparatus in plants at all developmental stages leading to significant reduction in agricultural productivity. Reduction in photosynthesis is the primary response that is observed in all glycophytic plants during salt stress. Employment of salt-tolerant plant growth-promoting bacteria (PGPB) is an economical and viable approach for the remediation of saline soils and improvement of plant growth. The current study is aimed towards investigating the growth patterns and photosynthetic responses of rice and tomato plants upon inoculation with halotolerant PGPB *Staphylococcus sciuri* ET101 under salt stress conditions. Tomato and rice plants inoculated with PGPB showed increased growth rate and stimulated root growth, along with higher transpiration rates (*E*), stomatal conductance (*g*_*s*_), and intracellular CO_2_ accumulation (Ci). Additionally, correlation of relative water content (RWC) to electrolyte leakage (EL) in tomato and rice plants showed decreased EL in inoculated plants during salt stress conditions, along with higher proline and glycine betaine content. Energy dissipation by non-photochemical quenching (NPQ) and increased photorespiration of 179.47% in tomato and 264.14% in rice plants were observed in uninoculated plants subjected to salinity stress. Furthermore, reduced photorespiration with improved salinity tolerance is observed in inoculated plants. The higher rates of photosynthesis in inoculated plants during salt stress were accompanied by increased quantum efficiency (ΦPSII) and maximum quantum yield (*F*_*v*_/*F*_*m*_) of photosystem II. Furthermore, inoculated plants showed increased carboxylation efficiency of RuBisCO, along with higher photosynthetic electron transport rate (ETR) (*J*) during salinity stress. Although the total cellular ATP levels are drastically affected by salt stress in tomato and rice plants along with increased reactive oxygen species (ROS) accumulation, the restoration of cellular ATP levels in leaves of inoculated plants along with decreased ROS accumulation suggests the protective role of PGPB. Our results reveal the beneficial role of *S. sciuri* ET101 in protection of photosynthesis and amelioration of salinity stress responses in rice and tomato plants.

## Introduction

Salinity stress is the major environmental problem all over the world due to which the cultivable land area is decreased, and drastic reduction in root length, biomass, and growth is observed causing a decline in crop yields (Deinlein et al., 2014; Janda et al., 2016; Kumar et al., 2020). The limitation of plant growth under salinity conditions is primarily due to reduction in photosynthesis rate and high intracellular accumulation of Na^+^ ions, which interfere with various physiological processes (Assaha et al., 2017; Analin et al., 2020). Decrease in net photosynthetic rate is associated with decreased availability of CO_2_ as a result of diffusion limitations and decrease in the contents of photosynthetic pigments (Tholen and Zhu, 2011). Decrease in intracellular CO_2_ concentration and photosynthesis during salinity stress due to stomatal closure leads to lesser availability of CO_2_ to the RuBisCO enzyme binding, thereby enhancing the rate of photorespiration (Igamberdiev, 2015). Salinity stress-induced accumulation of excess salts is known to affect the function of photosystems by modulating the photosynthetic proteins in chloroplasts causing irreversible damage to the photosynthetic apparatus at all developmental stages in glycophytic plants (Sun et al., 2016; Li et al., 2019; Rodríguez-Ortega et al., 2019; Zhang et al., 2020). Adaptation of plants to salinity stress involves modification of complex physiological traits, metabolic pathways, and gene networks (Nounjan et al., 2012; Gupta and Huang, 2014; Ghorbani et al., 2018; Kumar et al., 2020). The key photosynthetic processes including RuBisCO enzyme activity, ATP generation, electron transport rate (ETR), and efficiency of light capture in the photosystems are seriously affected by salt stress (Chaves et al., 2009). Soil oxygen deficiency due to osmotic effect and Na^+^/Cl^–^ toxicity is also observed in plants exposed to salinity stress (Barrett-Lennard and Shabala, 2013). The prevalence of hypoxia/anoxia conditions in root zone due to hindrance of O_2_ diffusion in soil leads to reduction of ATP formation and diminished growth (Kozlowski, 1997; Barrett-Lennard, 2003). The damage to the photosynthetic apparatus caused by salinity is reflected by change in chlorophyll a (Chl a) fluorescence parameters, photorespiration, and carboxylation/oxygenation efficiency (Pieters et al., 2003). Plants protect themselves against photodamage by increasing the non-photochemical quenching (NPQ) energy dissipation, thereby reducing the relative quantum efficiency of PSII (ΦPSII) to maintain adequate balance between the photosynthetic electron transport and carbon metabolism. The downregulation of the photochemical linear electron transport during salt stress conditions limits the oxidative stress, and increased cyclic electron flow increases the photoprotective energy dissipation (Stepien and Johnson, 2009).

A potential and conventional strategy to minimize the soil salinization is microbial-assisted amelioration of salt-induced damage (Prittesh et al., 2020). Among the microbiota, a promising perspective to improve plant salinity tolerance is involvement of halotolerant plant growth-promoting bacteria (PGPB) on to salt-sensitive plant species (Numan et al., 2018; Bakka and Challabathula, 2020). The main objective of using halotolerant PGPB is to increase plant growth, development rate, yield, and increased tolerance toward salinity. Many reports suggested the involvement of PGPB for ameliorating the abiotic stress-induced damages in plants. Isolation and characterization of potential bacteria with inherent capability of salt tolerance will be useful for inoculation to plants to increase crop productivity in saline regions (Ahmad et al., 2013; Kang et al., 2019; Bakka and Challabathula, 2020; Prittesh et al., 2020). The genus *Staphylococcus* has many species, which have been isolated from diverse environments and characterized as effective halotolerant and plant growth-promoting rhizobacteria (Nanjani and Soni, 2014; Shahid et al., 2019).

Tomato and rice are the two most important crops worldwide that serve as excellent model systems to understand their physiological changes when exposed to abiotic stress factors. The adjustment in photosynthetic process is a protective mechanism adapted by plants to tolerate salt stress (Parida and Das, 2015). However, the adjustment of plant photosynthetic processes due to the inoculation of bacteria under salt stress is rarely known. Whether the inoculation of halotolerant bacteria modulates the sensitivity of photosynthesis process to salt stress aiding in the improvement of plant stress tolerance is still unclear. Thus, the situation warrants for better understanding of photosynthesis process during salinity stress with bacterial inoculation.

In this article, we present data on the effects of salinity stress on photosynthesis in tomato (*Lycoperiscon esculentum* L.) and rice (*Oryza sativa* L.) under the influence of halotolerant bacteria, *Staphylococcus sciuri* ET101. Our study is focused on the physiological changes at the level of photosynthesis and photorespiration processes affected by different levels of salinity stress. We used a moderately salt-sensitive tomato and salt-sensitive rice plants to observe the physiological changes that can be attributed to acclimation by salinity stress in the presence of halotolerant, plant growth-promoting bacteria, *S. sciuri* ET101. Based on the results, we discuss the role of interplay between the carboxylation and oxygenation efficiency of RuBisCO as a possible factor for stress mitigation by the influence of bacterial inoculation.

## Materials and Methods

### Isolation and Characterization of Halotolerant Bacterial Isolate ET101

The bacterial isolate ET101 was isolated from the rhizosphere of common glasswort (*Salicornia europaea* L.) grown in the sandy soil of salt pan areas of Tuticorin district, Tamil Nadu, India. The 15-cm deeper bulk soil from the soil surface and soil associated with plant roots was used for the isolation of halotolerant bacteria. The isolation was carried by serial dilution plate technique followed by pure culture technique in high salt (2.5 M NaCl) supplemented LB agar medium. The preliminary characterization of the bacterial isolate ET101 based on physiological and biochemical characteristics was carried out according to Bergey’s Manual of Determinative Bacteriology (Holt et al., 1994). The molecular characterization of the ET101 isolate was carried out using colony polymerase chain reaction (PCR). Amplification of 16S rDNA gene was performed by using universal primers, 27F (5′-AGAGTTTGATCCTGGTCAGAACGCT-3′) and 1492R (5′-TACGGCTACCTTGTTACGACTTCACCCC-3′) (Weisburg et al., 1991). The PCR products were purified (PCR purification kit, Qiagen), and further sequencing of the amplicons was performed (Xcelris Genomic Services Pvt. Ltd., India). The bacterial isolate ET101 was screened for a wide array of PGP traits growing at different NaCl concentrations (0.08, 1.35, 1.7, 2.05, and 2.5 M). The production of indole-3-acetic acid (IAA) supplemented with or without 0.05% L-tryptophan was determined using the method of Gordon and Weber (1951). The quantitative estimation of gibberellic acid (GA) production was carried out by the method of Borrow et al. (1955). The assay of 1-aminocyclopropane-1-carboxylic acid deaminase (ACC deaminase) activity was determined by estimating the release α-ketobutyrate by the method of Honma and Shimomura (1978) and ammonia production by the method of Cappuccino and Sherman (1992).

### Plant Material, Bacterial Inoculation, Salt Stress Treatment, and Root Colonization

Tomato (*L. esculentum* cv. PKM-1) and rice (*O. sativa* cv. Aiswarya) seeds procured from Sri Venkateswara Agro Store, Tiruchirappalli, Tamil Nadu, India, and Regional Agricultural Research Station, Pattambi, Kerala, India, were surface sterilized using 0.4% sodium hypochlorite for 2 min followed by 70% ethanol for 2 min and thoroughly washed with sterile deionized water. For inoculum preparation, the bacterial isolate was grown in LB broth for 24 h at 30°C. After centrifugation, the obtained biomass was washed with phosphate-buffered saline (PBS) for three times and finally adjusted the absorbance of 0.8 at 600 nm corresponding to 10^8^ colony-forming units (CFU) mL^–1^ with sterile water. To ensure inoculation of sufficient bacterial cells per plant, bacterial inoculation is done to seeds and the plants germinated from the inoculated seeds. For seed inoculation, the surface sterilized seeds were kept immersed in the isolated bacterial suspension (10^8^CFU mL^–1^) overnight with mild shaking using bacteriological shaker incubator. The bacterial-treated seeds were sown for germination in sterile soil-containing pots to obtain plantlets of 15 days. Equally grown plantlets were transferred to the plastic pots (500-mL capacity for tomato and 200 mL for rice) containing sterile potted soil mixture. The potted soil mixture consisted of three parts of red soil, one part of soilrite, one part of vermiculite, and one part of perlite for growing tomato and rice plants. While the asbestos-free vermiculite is composed of SiO_2_ (40%), AlO_3_ (18%), FeO_3_ (11%), K_2_O (5%), MgO (13%), and Na_2_O (1%) and trace amounts of P_2_O_5_, C_4_O_3_, MgO_4_, the soilrite mix TC is composed of 75% Irish peat moss and 25% horticulture grade expanded perlite having pH range between 5.0 and 6.5. Perlite is primarily composed of silicon (Si) and does not have significant nutrient content. The red soil was purchased from the local vendor mainly used for growing horticultural plants and soilrite, vermiculite, and perlite were purchased from Keltech Energies Ltd., India. The texture of the red soil is fine loamy with dark red to brown color and with moderate porous nature. The soil mixtures procured from the same vendors were used throughout the study. The transferred plantlets were kept in observation for healthy growth up to 7 days. For bacterial inoculation in plants, the tomato and rice plants were inoculated with ET101 culture using the method of soil drenching, and the plant growth promotion by bacterial inoculation was evaluated. Each treated pot was inoculated with 5 mL of bacterial inoculum for 3 days once, whereas the control plants were treated with sterile water. For imposing salinity stress to plants, the initial soil water content in pots was equally maintained at uniform levels in both the plants with or without ET101 inoculation. The effect of salinity stress in uninoculated and inoculated plants was evaluated by irrigating the plants with different concentrations of NaCl solution (0 mM [S0], 200 mM [S2], and 400 mM [S4]) with 1/10th volume (50 mL for tomato plants and 20 mL for rice plants) of respective pots for every 2 days. The stress treatments were conducted for 5 days to rice plants and 10 days to tomato plants. Plants were grown in a growth chamber with artificial light provided by fluorescent tubes at 12:12-h light–dark period with the incident Photosynthetically Active Radiation (PAR) at leaf level of 140 μmol photons m^–2^ s^–1^ light intensity in controlled laboratory conditions. The plants were assessed based on phenotypic and physiological changes caused by the bacterial inoculation upon salinity stress. Temperature ranged between 22°C at night and 28°C during the light period. The changes in growth and stress tolerance were measured after the salinity stress treatment of 5 days to rice plants and 10 days to tomato plants. Root colonization by bacterial isolate ET101 was determined according to the protocol of Hossain et al. (2008). The root-associated soil, the surface of plants roots, and the homogenized root tissues were used as sources for enumeration of bacterial concentration in inoculated plants. The roots were thoroughly washed with running tap water to remove adhering soil particles and then were rinsed with sterile distilled water and surface sterilized using 70% ethanol and blotted to dryness. Roots were homogenized in PBS using sterile mortar pestle. Serial dilutions were prepared on LB plates containing 8% NaCl concentration, and the number of CFUs was determined after 24 h of incubation at 28 ± 2°C (Supplementary Figure S6).

### Measurement of Leaf Water Status, Electrolyte Leakage, and Osmolyte Production

The relative water content (RWC) and electrolyte leakage (EL) were measured in the fresh, fully developed leaves of the plant. RWC was determined using the equation previously described by Teulat et al. (2003). RWC (%) = (FW – DW)/(TW – DW) × 100, where FW is fresh weight; TW, turgid weight; and DW, dry weight of the leaf used. The leaves were harvested after prior treatments from the plants, and the fresh weights were measured using an electronic balance. After the fresh weight measurements, the leaves were immersed in Petri dishes containing sterile distilled water for overnight at room temperature. The turgid weight was determined by surface drying of the turgid leaves by using absorbent paper and measuring the leaf weight. For measuring the dry weight, the leaves were oven dried at 80°C overnight and weighed. For the measurement of EL, the leaves were cut, and discs were transferred into test tubes containing 25 mL of distilled water. The test tubes were incubated for 8 h and vortexed occasionally for 10 s, and the electrical conductivity (EC_1_) of the solution was measured using Systronics conductivity meter 304 at temperature of 25 ± 2°C in 2-μS range. Finally, the solution inside the test tubes was boiled for 20 min, and the electrical conductivity (EC_2_) was measured again. Electrolytic leakage percentage was calculated by the equation of Dionisio-Sese and Tobita (1998). Distilled water served as blank. EL (%) = EC_1_/EC_2_ × 100. Ninhydrin was used to determine the proline contents of plants (Bates et al., 1973). The proline content in each treatment samples was determined by measuring the absorbance at 520 nm. The amount of proline was extrapolated using a standard curve prepared with L-proline and expressed in μg g^–1^. The production of glycine betaine by the plants during different salt stress was estimated using the method of Grieve and Grattan (1983). The values were extrapolated using betaine hydrochloride as standard and expressed as μg L^–1^.

### Measurement Photosynthetic Pigments

The chlorophyll was extracted from the fresh and fully expanded leaf samples (100 mg) with 80% acetone at 4°C under dark (Moran and Porath, 1980). Chlorophyll was estimated spectrophotometrically by measuring the absorbance of Chl a, chlorophyll b (Chl b), and carotenoid at 663, 645, and 480 nm, respectively, by using the equation of Arnon (1949): Chl a (mg/g^–1^) = [12.7 (Ab663) – 2.69 (Ab645)] ^∗^ *V*/1,000 ^∗^ *W*; Chl b (mg/g^–1^) = [22.9 (Ab645) – 4.68 (Ab663)] ^∗^ *V*/1,000 ^∗^ *W*; Total Chlorophyll (mg/g^–1^) = [Chl a + Chl b]; Carotenoid (mg/g^–1^) = [Ab480 + (0.114 ^∗^ Ab663) – (0.638 ^∗^ Ab645)] ^∗^ *V*/1,000 ^∗^ *W*, where *V* is final volume of chlorophyll extracted in 80% acetone; and W, fresh weight of the leaf used.

### Measurement of Gas Exchange Parameters

The *in vivo* measurements of net photosynthesis rate (*P*_*N*_), stomatal conductance (*g*_*s*_), and transpiration (*E*) of the plants subjected to salt treatment with or without bacterial inoculation were done as light-response curves using a portable open-flow gas exchange system (LI-6400XT, LI-COR, Lincoln, NE, United States) at the levels of photosynthetic photon flux density (PPFD) of 30, 50, 75, 100, 150, 200, 500, 750, 1,000, and 1,500 μmol photons m^–2^ s^–1^ in a decreasing order at 25°C and the CO_2_ concentration of 400 μmol CO_2_ mol^–1^ air, ambient air humidity, and leaf temperature of 25°C. In addition to CO_2_ assimilation rate, the value of ratio of CO_2_ assimilation (*P*_*N*_) to substomatal internal CO_2_ content (Ci) is shown to recognize whether stomatal or non-stomatal limitation of photosynthesis is present.

### Measurement of Chlorophyll Fluorescence Parameters

The Chl *a* fluorescence parameters were determined by using a fluorescence chamber head (LI-6400-40, LI-6400XT, LI-COR, Lincoln, NE, United States) integrated with the portable open-flow gas exchange system. While weak modulated measuring beams (0.03 μmol m^–2^ s^–1^) were used for illuminating the dark-adapted leaves (20 min) to obtain the initial fluorescence (*F*_0_), the saturating white light pulses of 8,000 μmol photons m^–2^ s^–1^ were applied for 0.8 s to ensure maximum fluorescence emissions (*F*_*m*_). In light-adapted leaves, the steady-state fluorescence yield (*F*_*s*_) was measured following a saturating white light pulse (8,000 μmol m^–2^ s^–1^, 0.8 s) that was applied to achieve the light-adapted maximum fluorescence (*F*${}_{m}^{\prime }$). The actinic light was then turned off, and far-red illumination was applied (2 μmol m^–2^ s^–1^) to measure the light-adapted initial fluorescence (F${}_{0}^{\prime }$). The NPQ was calculated as NPQ = (*F*_*m*_/*F*_*m*_*’*) – 1. The actual PSII quantum yield was computed as *Φ*_*PSII*_ = (*F*${}_{m}^{\prime }$ – *F*_*s*_)/*F*${}_{m}^{\prime }$ from which the apparent ETR (*J*) was calculated as *J* = *Φ*_*PSII*_^∗^ PPFD ^∗^ *f* ^∗^ α, where PPFD was 1,500 μmol m^–2^ s^–1^, *f* is a factor that accounts for the partitioning of energy between the PSI and PSII and is assumed to be 0.5 (indicating that the excitation energy is distributed equally between the two photosystems), and α is the leaf absorbance by the photosynthetic tissues and is assumed to be 0.87 (Maxwell and Johnson, 2000). All the measurements were determined on the middle leaf attached to each plant (three leaves per treatment). Photorespiration (*P*_*R*_) was estimated as 1/12 [*J* – 4 × (*P*_*N*_ + *R*_*D*_)] according to Valentini et al. (1995), where *P*_*N*_ is net photosynthesis rate and *R*_*D*_ is respiration rate in dark. Gas exchange was also measured in dark-adapted leaves after overnight dark incubation to obtain dark respiration rate (*R*_*D*_) (μmol CO_2_ released m^–2^ s^–1^). The leaves were dark adapted for 20 min in the chamber head for measuring the dark respiratory rates (CO_2_ efflux) using the portable photosynthesis system (LI-6400 XT; LI-COR Inc., Lincoln, NE, United States) in darkness. The CO_2_ efflux rates were measured for 20 min with 1-min interval and were calculated as the difference between the ambient CO_2_ and sample CO_2_. The CO_2_ concentration in the chamber ambient air (Ca) was maintained at 400 μmol mol^–1^.

According to Epron et al. (1995), *J* was divided into two components: *J*_*f*_ = *J*_*c*_ + *J*_*o*_.*J*_*c*_ is the fraction of *J*_*f*_ used for CO_2_ assimilation (*J*_*c*_ = 1/3 [*J* + 8(*P*_*N*_ + *R*_*L*_)]), and *J*_*o*_ is the fraction of *J*_*f*_ used for photorespiration (*J*_*o*_ = 2/3 [*J* – 4 (*P*_*N*_ + *R*_*L*_)]). The *J*_*o*_/*J*_*c*_ indicates the ratio of linear electron transport involved in oxygenation to the carboxylation. The calculated values of *J*_*o*_ and *J*_*c*_ were used to depict the changes in switching over of linear ETR from carboxylation to oxygenation. This approach assumes that all the reducing power generated by electron transport chain is used for photosynthesis and photorespiration, and Chl fluorescence gives reliable estimation of the quantum yield of electron transport. The rate of linear transport of electron involved in both carboxylation and oxygenation of RuBisCO was calculated according to Harley et al. (1992) as follows: *J*_*g*_ = 4(*P*_*N*_ + *R*_*L*_)(Ci + 2*I*^–*^)(Ci – *I*^–*^), where Ci represents intercellular content of CO_2_, *R*_*L*_ represents mitochondrial respiration in light (Yin et al., 2009), and *I*^–*^ represents CO_2_ compensation point measured in the absence of respiration using A-Ci curve. The rates of carboxylation (*V*_*c*_) and oxygenation (*V*_*o*_) of RuBisCO were calculated as follows: *V*_*c*_ = 1/6[*J*_*f*_/2 + 4 (*P*_*N*_ + *R*_*L*_)] and *V*_*o*_ = 1/6[*J*_*f*_ – 4 (*P*_*N*_ + *R*_*L*_)], respectively (von Caemmerer, 2000).

### Estimation of Adenylates

The estimation of total cellular adenylates was done by the method of Padmasree and Raghavendra (1999) and Dinakar et al. (2016). After the salt stress treatment, the 100 mg of fresh leaf samples was ground in liquid nitrogen using 3% HClO_4_ (vol/vol) and centrifuged at 7,000 *g* for 10 min. The supernatants were neutralized using 150 mM triethanolamine (TEA) and incubated on ice for 30 min prior to centrifugation of 7,000 *g* for 10 min. The clear supernatant was used for estimation of ATP and ADP. The assay medium for ATP contained 150 mM TEA buffer (pH 7.5), 10 mM MgCl_2_, 0.5 mM NADP, and 100 μL of neutralized sample. Glucose-6-phosphate dehydrogenase (0.023 μkat) (E.C. 1.1.1.49) was added to consume internal Glc-6-P levels. ATP levels were monitored by following the net increase in absorbance at 340 nm after addition of 10 mM glucose and 0.047 μkat hexokinase (E.C. 2.7.1.1). The assay medium for the assay of ADP contained 150 mM Tris-HCl pH 8.1, 7.5 mM MgCl_2_, 0.08 mM NADH, 2 mM phosphoenol pyruvic acid, 100 μL of neutralized sample, 0.046 μkat lactate dehydrogenase (E.C. 1.1.1.27), and 0.067 μkat pyruvate kinase (PK, E.C. 2.7.1.40). The content of ADP was calculated from the net decrease in absorbance at 340 nm after the addition of PK.

### *In vivo* Localization of Reactive Oxygen Species and Cell Damage in Leaf Tissues

Histochemical staining using 3,3′-diaminobenzidine (DAB) tetrahydrochloride and nitroblue tetrazolium (NBT, Sigma) was used to study *in vivo* localization of reactive oxygen species (ROS) (H_2_O_2_ and superoxide) in uninoculated and inoculated leaves of control and NaCl-treated plants. The leaves of both the plants were vacuum infiltrated by freshly prepared DAB solution (1 mg/mL) and NBT (1 mg/mL) in 10 mM potassium phosphate buffer (pH 7.8). The infiltrated leaves were kept in the dark overnight and placed under continuous light (300 μmol m^–2^ s^–1^) at 25°C for 8 h. The stained leaves were bleached in a warm destaining solution [methanol: acetic acid: glycerol (3:2:1)] and fixed using a fixative reagent [methanol: deionized water: glycerol (5:4:1)] (Rangani et al., 2016). The tissue damage in leaves was observed by trypan blue staining method. The freshly harvested leaves were incubated overnight in lacto phenol-trypan blue solution (10 mL lactic acid, 10 mL glycerol, 10 g phenol, and 10 mg trypan blue dissolved in 10 mL distilled water) (Koch and Slusarenko, 1990). Stained leaves were then boiled for 1 min and then decolorized in 95% hot ethanol solution. The images of leaf tissues were obtained using a Canon Lide 120 Scanner under uniform white background.

### Statistical Analysis

The statistical analysis was carried out using Sigmaplot v. 14.0 (Systat Software Inc.). The values obtained from three biological and three technical replicates were used for calculating the mean. The difference between the means of rates in the leaves of uninoculated and inoculated plants subjected to salinity stress was made by using analysis of variance. Multiple pairwise comparisons between different samples using Holm–Sidak test at a significance level of (*P* < 0.001) were used.

## Results

### Characterization of ET101 Isolate

**FIGURE 1:**
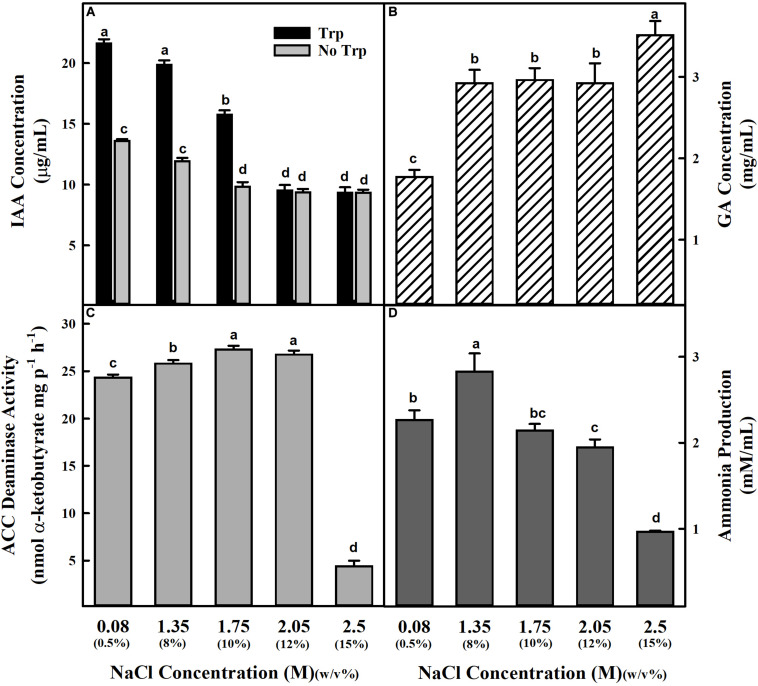
Estimation of IAA, GA, ACC deaminase activity, and ammonia production by *Staphylococcus sciuri* ET101 isolate at different concentrations of NaCl. **(A)** The IAA production by the *S. sciuri* isolate ET101 at different NaCl concentrations with or without 0.5% L-tryptophan supplementation. **(B)** The quantitative estimation of GA production by the *S. sciuri* isolate ET101 at different NaCl concentrations. **(C)** The ACC deaminase activity by the *S. sciuri* isolate ET101 at different NaCl concentrations with 3 mM ACC. **(D)** The ammonia production by the *S. sciuri* isolate ET101 at different NaCl concentrations. Bars represent mean ± SE of three independent replicates. Different letters on bars indicate significant difference between the salt treatments and bacterial inoculation (ANOVA; *P* < 0.001). Other details are mentioned in Section “Materials and Methods.”

The physiological, biochemical, and molecular analysis revealed the isolated halophilic isolate ET101 as *S. sciuri.* The sequence of the isolate ET101 was submitted in GenBank with accession number MN960659. Phylogenetic tree analysis showed *S. sciuri* strain Dc-04 as the nearest homolog with 99% sequence similarity (Supplementary Figure S1). *S. sciuri* ET101 was further characterized for the production of various plant growth-promoting substances in the absence and presence of NaCl. Isolate ET101 produced substantial amount of IAA under *in vitro* conditions in the presence and absence of tryptophan, suggesting that it could synthesize IAA through tryptophan independent and dependent pathways. However, IAA accumulation is decreased in the absence of tryptophan. Additionally, upon treatment with increasing NaCl concentrations of 2.05 and 2.5 M, a noticeable decrease in IAA production (54.36%) was observed (Figure 1A). High amount of GA is produced by ET101 isolate irrespective of salt stress treatment. An increase in GA production (64.84–98.36%) was observed in bacteria treated with higher concentrations of NaCl (2.05 and 2.5 M) (Figure 1B). The isolate ET101 also exhibited ACC deaminase activity by producing α-ketobutyrate (26.74 ± 0.36 nmol/mg protein) after 96 h of incubation in the presence of 2.05 M NaCl (Figure 1C). However, at higher concentrations of NaCl (2.5 M), the amount of α-ketobutyrate production decreased by > 81.93%. Although ET101 produced higher amount of ammonia, 2.26 ± 0.113 μM/10^8^ CFU after 96 h of incubation (Figure 1D), with the increase in concentration of NaCl, a progressive decrease in ammonia production was observed.

### Effect of Salt Stress, Water Relations, and Osmolytes Production in Uninoculated and Inoculated Tomato and Rice Plants

The uninoculated tomato and rice plants grown under different concentrations of NaCl (S0-0 mM NaCl, S2-200 mM NaCl, and S4-400 mM NaCl) exhibited a noticeable growth inhibition. The shoot length, root length, and biomass of plants decreased with increasing NaCl concentrations (Supplementary Figures S2,S3). In uninoculated rice plants treated with 200 mM NaCl (C2), while the percent decrease was 21.94% for shoot length, 8.62% for root length, and 11.19% for biomass; the plants treated with 400 mM NaCl (C4) showed decreases of 31.98, 32.45, and 15.59%, respectively (Supplementary Figure S5). Compared to uninoculated rice plants, increases in shoot length, root length, and biomass by 30.64, 16.44, and 1.48% were observed in ET101-inoculated rice plants (E0) without salinity stress treatment. Similarly, increases in shoot length (8.75%), root length (73.02%), and biomass (114.79%) were observed in inoculated tomato plants under control conditions (E0 treatment). Isolate ET101 induced remarkable increases in plant biomass under 200 mM NaCl (E2) and 400 mM NaCl (E4) treatments by 69.11 and 76.44%, respectively (Supplementary Figure S5).

**FIGURE 2:**
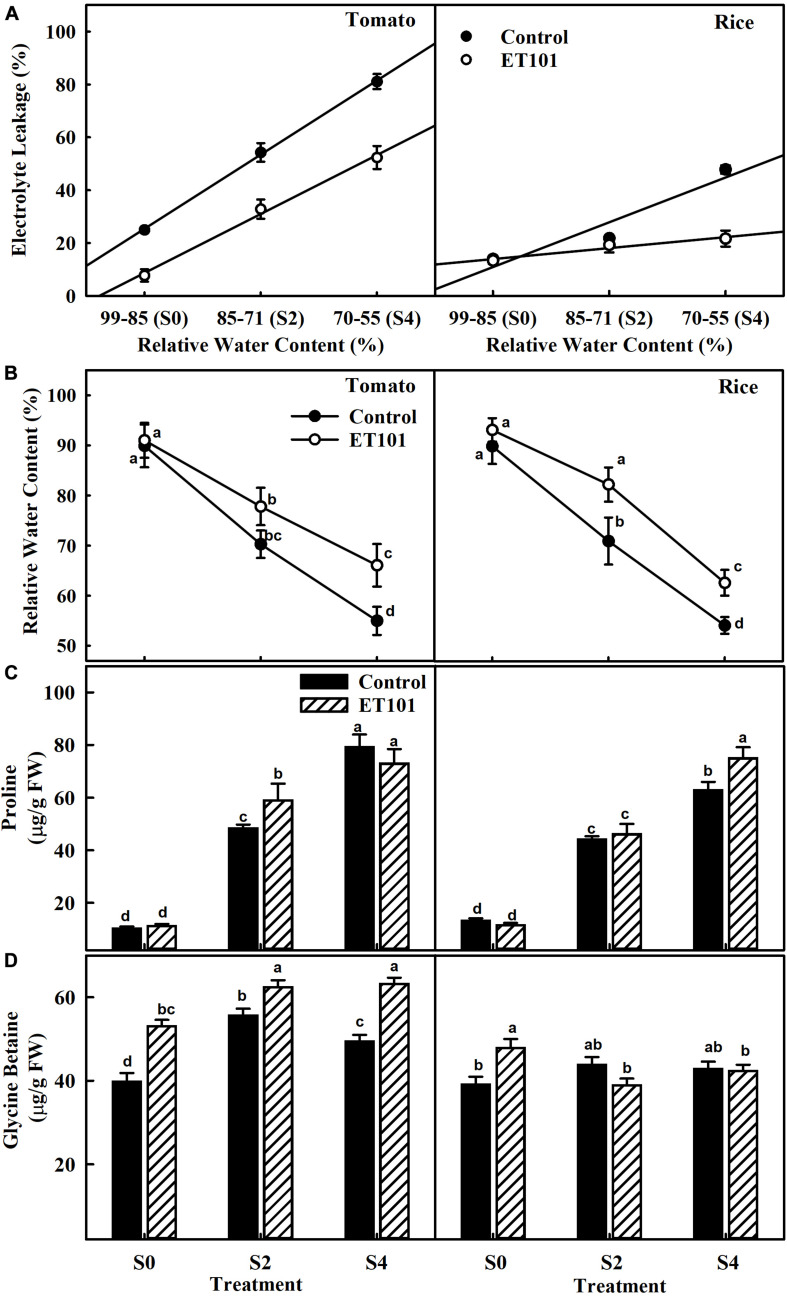
Changes in relative water content, electrolyte leakage, and osmolyte production in leaves of uninoculated and inoculated tomato and rice plants subjected to salinity stress conditions. **(A)** Effect of *Staphylococcus sciuri* isolate ET101 inoculation on leaf relative water content and electrolyte leakage of tomato and rice plants subjected to different salt stress treatments (S0: 0 mM NaCl, S2: 200 mM NaCl, S4: 400 mM NaCl). **(B)** Leaf relative water content of tomato and rice plants. **(C)** Effect of ET101 inoculation on proline accumulation of leaves of tomato and rice plants. **(D)** Effect of ET101 inoculation on glycine betaine accumulation in leaves of tomato and rice plants. The open symbols denote ET101-inoculated plants, whereas the closed symbols represent uninoculated tomato and rice plants. Different letters on bars indicate significant difference between the salt treatments and bacterial inoculation (ANOVA; *P* < 0.001). Other details are mentioned in Section “Materials and Methods.”

Significant increase in EL was observed in both uninoculated and inoculated plants of tomato and rice plants subjected to S2 and S4 salt stress. However, compared to uninoculated plants, the EL in inoculated plants was significantly lower (Figure 2A). While the RWCs in uninoculated tomato and rice plants were 89.10 and 96.20%, salinity stress led to a remarkable decrease in leaf water status in S2 and S4 stress-treated plants. The correlation of RWC to EL in tomato and rice plants shows decreased EL in inoculated plants during S2 and S4 stress conditions (Figure 2A). Remarkable increase in proline accumulation was observed in uninoculated and inoculated tomato and rice plants during S2 and S4 stress conditions. However, ET101-inoculated tomato and rice plants showed better RWC under control conditions and salt stress conditions (Figure 2B). Compared to uninoculated salt-stressed tomato plants, increases in RWC by 10.68 and 20.16% were observed in ET101-inoculated plants subjected to S2 and S4 salt stress conditions. Similarly, the ET101-inoculated rice plants also showed the lesser loss of water from leaf tissues and showed an increase in RWC by 14.91 and 9.12% at S2 and S4 salt stress treatments (Figure 2B). When compared to uninoculated tomato plants, although marginal non-significant decrease in proline content was observed in inoculated tomato plants subjected to S4 salt stress. While the ET101-inoculated rice plants showed marginal increase in proline content in S2 stress condition, a significant increase by 18.11% was observed under S4 stress condition. In ET101-inoculated tomato plants, compared to uninoculated plants at S2 stress condition, the increase in proline content was observed only under S2 stress condition (Figure 2C). Accumulation of glycine betaine was also observed in both tomato and rice plants in S2 and S4 salt stress condition. Compared to uninoculated plants, the amounts of glycine betaine were significantly increased, in tomato plants (33.52, 12.16, and 27.80%, respectively), whereas a marginal decrease (11.20 and 1.17%) was observed in rice plants inoculated with ET101 (Figure 2D).

### Measurement of Photosynthetic Pigments and Gas Exchange Parameters

**FIGURE 3:**
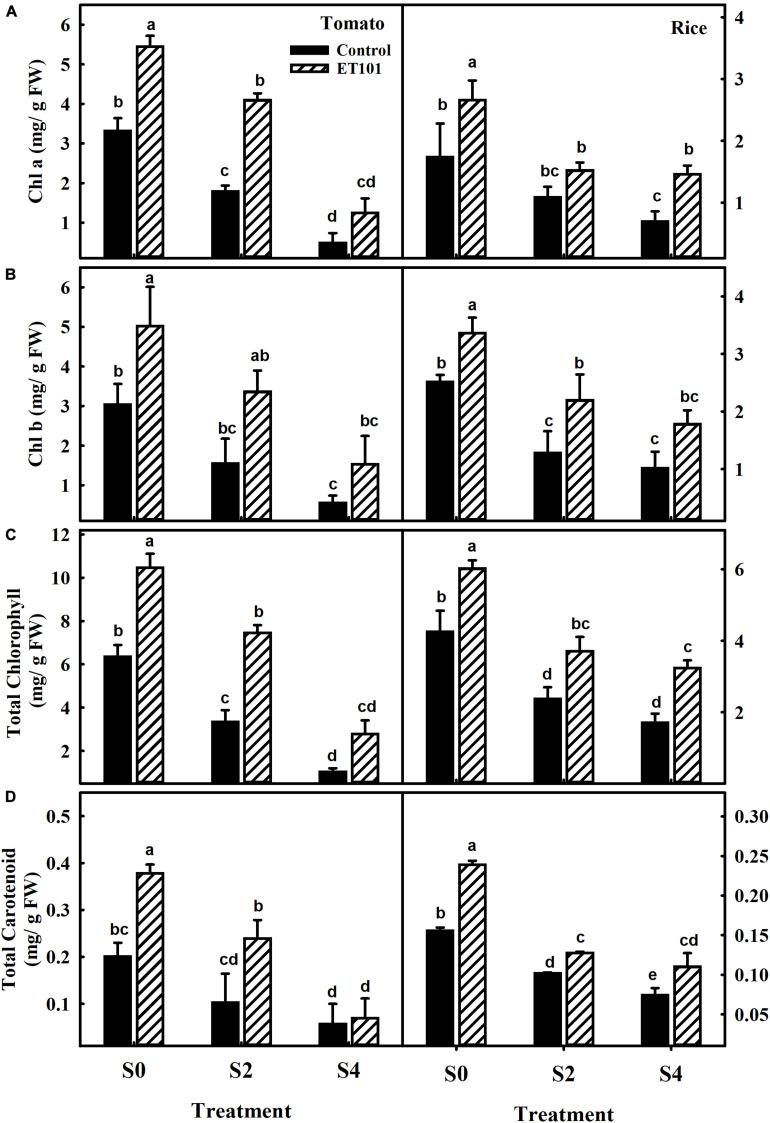
Changes in photosynthetic pigments and carotenoid content in leaves of uninoculated and inoculated tomato and rice plants. Changes in **(A)** chlorophyll a (Chl a), **(B)** chlorophyll b (Chl b), **(C)** total chlorophyll content, and **(D)** total carotenoid content in leaves of tomato and rice plants subjected to different salt treatments (S0: 0 mM NaCl, S2: 200 mM NaCl, and S4: 400 mM NaCl). Data represent the mean ± SE of triplicates. Different letters on bars indicate a significant difference between the salt treatments and bacterial inoculation (ANOVA; *P* < 0.001). Other details are mentioned in Section “Materials and Methods.”

**FIGURE 4:**
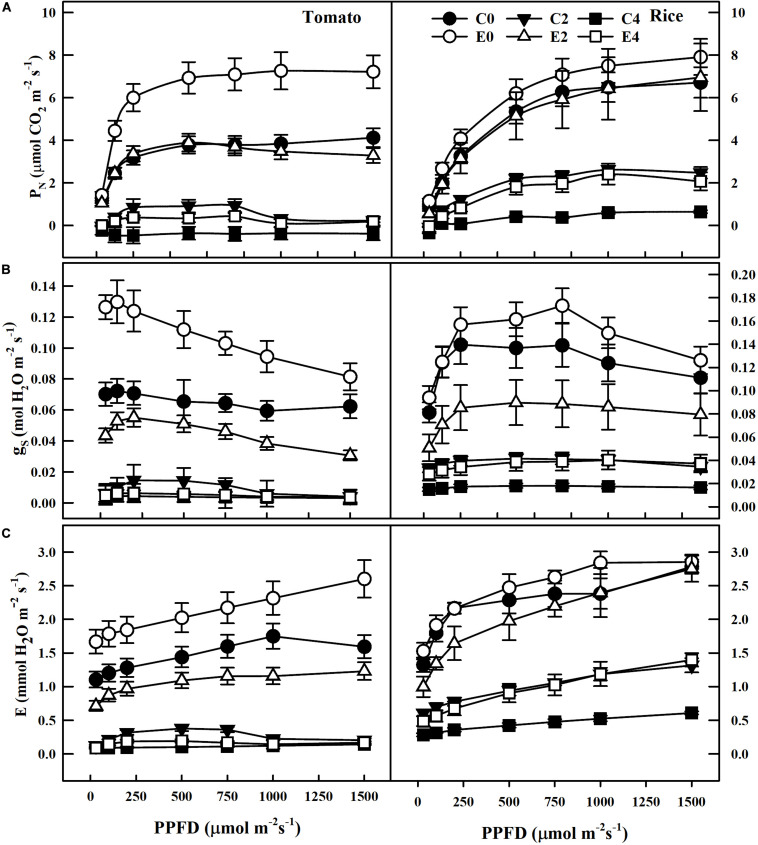
Changes in CO_2_ assimilation and gas exchange parameters in tomato and rice plants under different PPFD. **(A)** Effect of inoculation of ET101 isolate on CO_2_ assimilation (A_*CO2*_). **(B)** Effect of inoculation of ET101 isolate on stomatal conductance (g_*s*_). **(C)** Effect of inoculation of ET101 isolate on transpiration (*E*) in leaves of tomato and rice plants subjected to different salt stress treatments. Values represent mean of triplicates from sample size *n* = 6. The open symbols denote ET101-inoculated plants, whereas the closed symbols represent uninoculated tomato and rice plants (C0: control + 0 mM NaCl, C2: control + 200 mM NaCl, C4: control + 400 mM NaCl; E0: ET101 + 0 mM NaCl, E2: ET101 + 200 mM NaCl, E4: ET101 + 400 mM NaCl).

**FIGURE 5:**
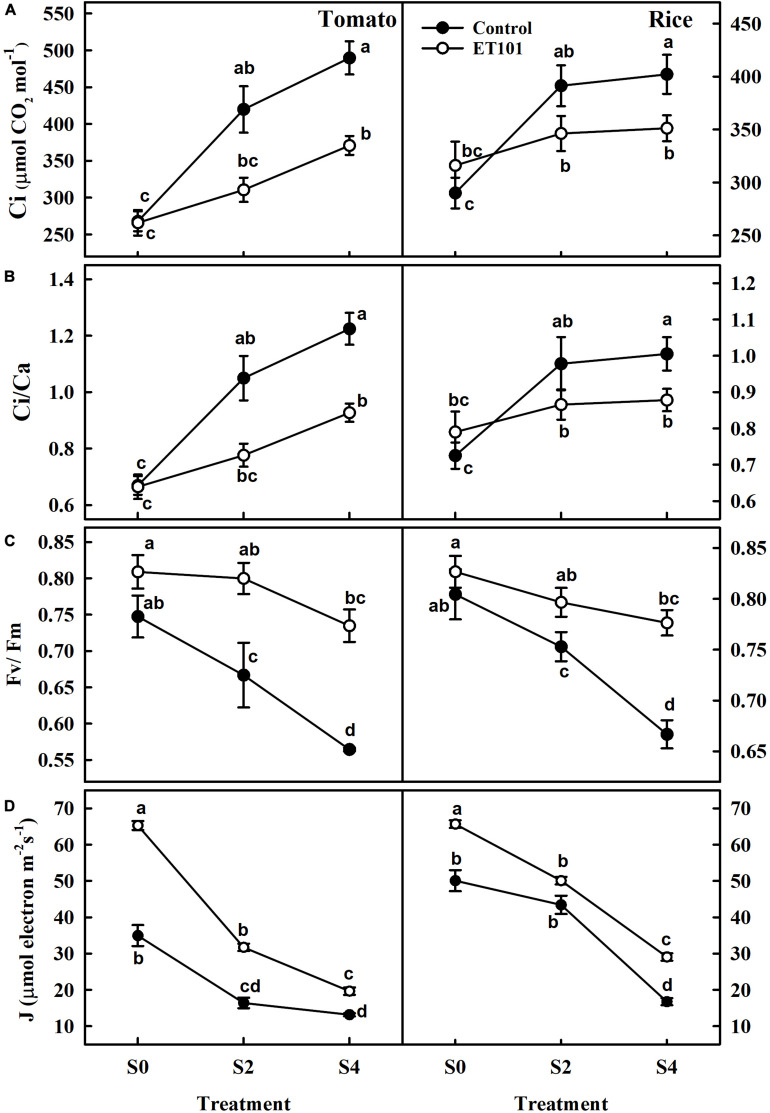
Changes in **(A)** intracellular CO_2_ concentration (Ci), **(B)** ratio of intercellular CO_2_ to ambient CO_2_ concentration (Ci/Ca), **(C)** maximum quantum yield (*F*_*v*_/*F*_*m*_), and **(D)** rate of electron transport rate in PSII (*J*) in tomato and rice plants subjected to different salinity treatments (S0: 0 mM NaCl, S2: 200 mM NaCl, and S4: 400 mM NaCl). The open symbols denote ET101-inoculated plants, whereas the closed symbols represent uninoculated tomato and rice plants. Values represent mean of triplicates from sample size *n* = 6. Different letters on bars indicate a significant difference between the salt treatments and bacterial inoculation (ANOVA; *P* < 0.001). Other details are mentioned in Section “Materials and Methods.”

Remarkable decreases in Chl a, Chl b, total chlorophyll, and carotenoid contents were observed in leaves of uninoculated tomato and rice plants during salt stress conditions (C0, C2, and C4). However, plants inoculated with ET101 isolate (E0, E2, and E4) showed significant increase in Chl a, Chl b, total chlorophyll, and carotenoid content under different salt stress conditions (Figures 3A–D). The salinity stress has considerable influence on the photosynthetic parameters such as net photosynthetic rate (*P*_*N*_), transpiration (*E*), and stomatal conductance (*g*_*s*_) (Figures 4A–C). Under all tested light conditions, salinity stress severely impacted the photosynthetic parameters. Drastic decrease in net photosynthetic rate, transpiration, and stomatal conductance was observed in both tomato and rice leaves subjected to S2 and S4 salinity stress. In uninoculated plants, the *P*_*N*_ rates gradually increased with increasing light intensities; however, upon imposition of salinity stress, the *P*_*N*_ rates were decreased to a greater extent in both C2 and C4 conditions at all given light intensities. However, inoculation of tomato and rice plants with ET101 isolate resulted in higher *P*_*N*_ rates than uninoculated plants grown under C0 (76.08% in tomato and 20.14% in rice), C2 (13.11 and 76.25%), and C4 (347.11 and 220.119%) conditions (Figure 4A). Similar trend has been observed in the transpiration rates (*E*) and stomatal conductance (*g*_*s*_). The decrease in transpiration rate (*E*) and stomatal conductance (*g*_*s*_) was higher in uninoculated plants than inoculated plants at all light intensities. The stomatal conductance (*g*_*s*_) in tomato E0 plants was significantly higher than in C0, whereas marginal difference was observed in the rice C0 and E0 plants. However, significant increase was attained between E2, E4, and C2, C4 plants of both tomato and rice varieties (Figures 4B,C). While the intercellular CO_2_ rates were significantly higher in uninoculated tomato and rice plants subjected to S2 and S4 salt stress conditions than inoculated plants, the ratio of intercellular CO_2_ to ambient (Ci/Ca), reflecting the relationship between *g*_*s*_ and non-stomatal capacity for photosynthesis was also significantly higher in uninoculated S2 and S4 treatments than ET101-inoculated tomato and rice plants (Figures 5A,B).

### Measurement Chlorophyll Fluorescence Parameters

The photosynthetic induction in dark-adapted leaves was monitored by saturation pulse method of chlorophyll fluorescence measured together with gaseous exchange. These measurements were used for the calculation of quenching parameters (Table 1). The maximum quantum efficiency of PSII photochemistry (*F*_*v*_/*F*_*m*_) in uninoculated tomato and rice plants at S2 and S4 stress treatments showed decreases of 6.40 and 10.8%, and 17.12 and 24.50% (Figure 5C). Although marginal increase in *F*_*v*_/*F*_*m*_ ratio was observed in inoculated plants than uninoculated plants at S0 (Control) in both tomato and rice plants, significant increase was observed at S2 and S4 stress treatments in ET101-inoculated plants. The ET101-inoculated plants exhibited marginal decrease in the *F*_*v*_/*F*_*m*_ ratio of approximately 1.12 and 3.63% in S2 and 9.17 and 3.65% in S4 salt stress treatment in tomato and rice plants, respectively (Figure 5C). Although ETRs (*J*) were significantly higher in ET101-inoculated tomato and rice leaves than uninoculated leaves at S0 treatment, the rates gradually declined in tomato and rice leaves under S2 and S4 salinity stress. However, under control and salinity stress conditions, the leaves of ET101-inoculated plants had higher ETR (*J*) than uninoculated control plants (Figure 5D).

**TABLE 1:** The chlorophyll fluorescence parameters derived from the saturation pulse analysis.

**Chlorophyll fluorescence parameters derived from the saturation pulse analysis**		
*F*, *F*′	Fluorescence emission from dark- or light-adapted leaf, respectively	
*F*_0_	Minimum fluorescence from dark-adapted leaf (PS II centers open)	
*F*_*m*_, *F*${}_{m}^{\prime }$	Maximum fluorescence from dark- or light-adapted leaf, respectively (PS II centers closed)	
*F*_*V*_ = *F*_*m*_ − *F*_0_	Maximum variable fluorescence from dark-adapted leaf	
*F*${}_{0}^{\prime }$	Minimum fluorescence from light-adapted leaf	
*F*_*s*_	Steady-state fluorescence at any light level	
*F*_*V*_/*F*_*M*_ = 1 – (*F*_*o*_/*F*_*m*_)	Estimated maximum quantum efficiency of PSII photochemistry	Kitajima and Butler, 1975; Krause and Weis, 1991; Schreiber et al., 1995
**Φ**_*PSII*_ = (*F*${}_{m}^{\prime }$ – *F*_*s*_)/*F*${}_{m}^{\prime }$	Estimated effective quantum yield of PSII photochemistry at given PAR	Genty et al., 1989
*J*_*PSII*_ = α_*II*_ * PAR * **Φ**_*PSII*_	Rate of linear electron transport in PSII at given PAR and portion of PAR absorbed by PSII (α_*II*_)	Bjorkman and Demmig, 1987; Genty et al., 1989
NPQ = (*F*_*m*_ – *F*${}_{m}^{\prime }$)/*F*${}_{m}^{\prime }$	Non-photochemical quenching of *F*_*m*_	Schreiber et al., 1988; Walters and Horton, 1991
*qP* = (*F*${}_{m}^{\prime }$ – *F*_*s*_)/(*F*${}_{m}^{\prime }$ – *F*${}_{0}^{\prime }$)	Coefficient of photochemical quenching based on the “puddle” model (i.e., unconnected PS II units)	Schreiber, 1986; Bjorkman and Demmig, 1987; Bilger and Bjorkman, 1990

**FIGURE 6:**
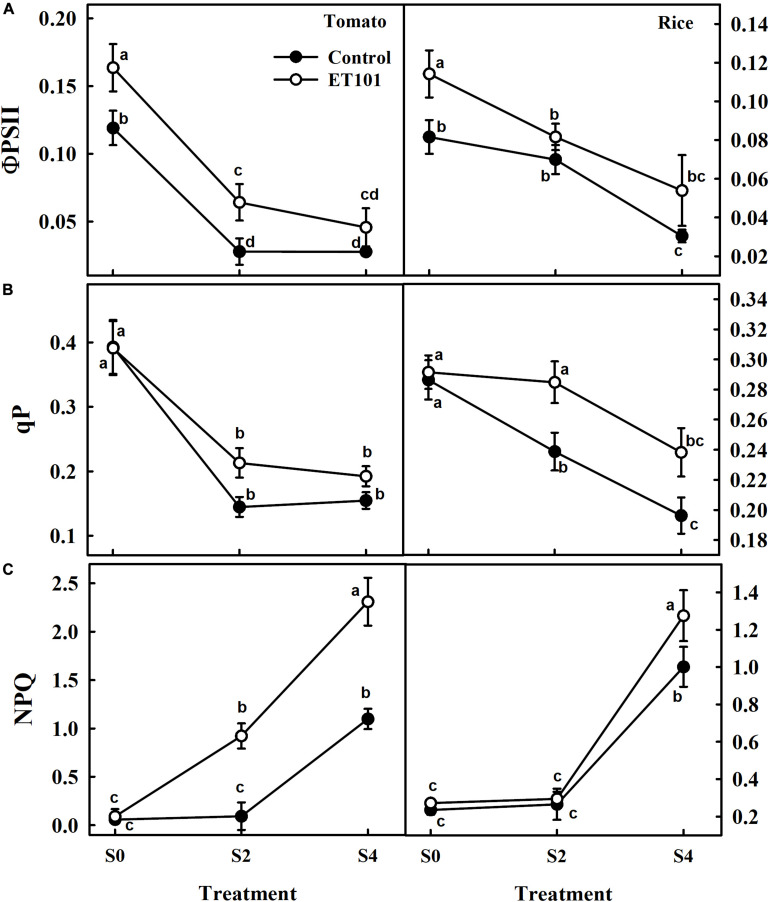
Changes in **(A)** quantum efficiency (ΦPSII), **(B)** photochemical quenching (*qP*), and **(C)** non-photochemical quenching (NPQ) in leaves of tomato and rice plants subjected to salinity stress (S0: 0 mM NaCl, S2: 200 mM NaCl, and S4: 400 mM NaCl). Values represent the mean of triplicates from sample size (*n* = 6). The open symbols denote ET101-inoculated plants, whereas the closed symbols represent uninoculated tomato and rice plants. Different letters indicate significant difference between the salt treatments and bacterial inoculation (ANOVA; *P* < 0.001). Other details are mentioned in Section “Materials and Methods.”

The estimated effective quantum yield of PSII (ΦPSII), although was higher in leaves of ET101-inoculated tomato and rice plants under control and salinity stress conditions, decreased with increasing salinity stress in both uninoculated and ET101-inoculated tomato and rice plants (Figure 6A). The inoculated tomato and rice plants showed higher ΦPSII rates than uninoculated plants. Compared to uninoculated plants, while the increase in ΦPSII rates in ET101-inoculated control plants was 14.46% in tomato and 39.89% in rice, the pronounced increase was observed in the leaves of inoculated plants under S2 (130.77% in tomato and 16.74% in rice) and S4 (65.49% in tomato and 77.38% in rice) stress conditions (Figure 6A). Although significant differences were not observed in the photochemical quenching (*qP*) rates between ET101-inoculated and uninoculated tomato and rice plants in S0 treatment, leaves of ET101-inoculated plants showed higher *qP* values of approximately 47.22 and 19.28%, and 24.26 and 21.44% in tomato and rice plants during S2 and S4 salinity stress conditions (Figure 6B). The uninoculated tomato and rice plants exposed to salinity stress conditions (S4) showed a significant increase in NPQ (Figure 6C). However, the higher increase was observed in tomato and rice plants grown under S2 and S4 salinity stress condition with ET101 inoculation. Inoculation with ET101 increased NPQ of plants exposed to S2 and S4 salinity conditions by 11.10 to 110.4% compared with respective controls (Figure 6C).

**FIGURE 7:**
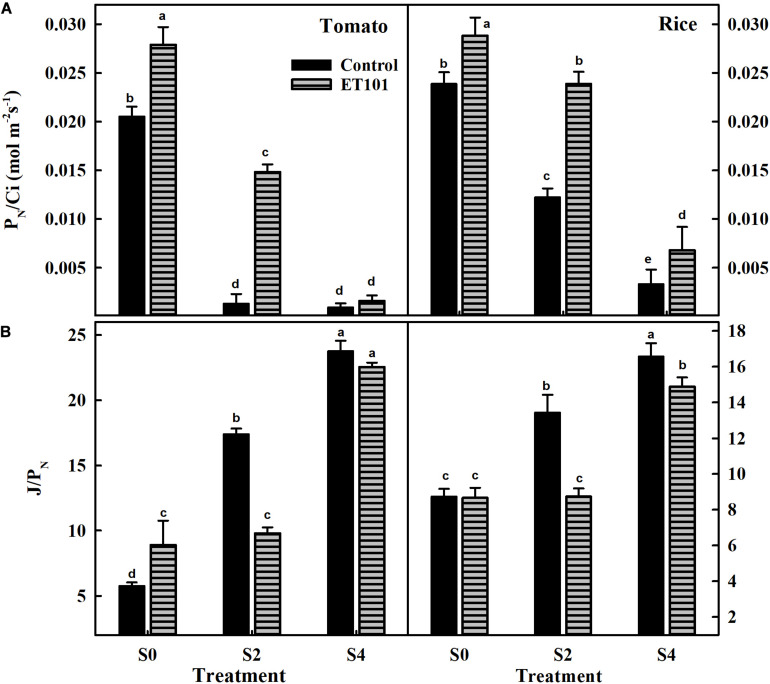
**(A)** The rate of apparent RuBisCO efficiency (*P*_*N*_/Ci) and **(B)** the ratio of electron transport and net photosynthesis (*J*/*P*_*N*_) of uninoculated and ET101-inoculated tomato and rice plants subjected to different salt stress treatments (S0: 0 mM NaCl, S2: 200 mM NaCl, S4: 400 mM NaCl). Bars represent mean ± SE of triplicates. Different letters indicate significant difference between the salt treatments and bacterial inoculation (ANOVA; *P* < 0.001). Other details are mentioned in Section “Materials and Methods.”

Net photosynthetic rates (*P*_*N*_) measured in leaves of ET101-inoculated tomato and rice plants were significantly higher than that of uninoculated plants in all treatments (Supplementary Figure S4). The apparent RuBisCO efficiency (*P*_*N*_/Ci) measured in uninoculated plants showed a significant reduction in S2 and S4 treatments in tomato and rice plants. The leaves of uninoculated plants showed decreases of 93.58 and 95.48% in tomato and 48.74 and 86.15% in rice during S2 and S4 salinity treatments. However, the leaves of ET101-inoculated plants showed lesser reduction of about 46.78 and 94.26% in tomato and 17.15 and 76.35% in rice plants, respectively (Figure 7A). The ratio of ETR and net photosynthesis (*J*/*P*_*N*_) ratio measured in uninoculated tomato and rice plants showed incremental increase in S2 and S4 treatments (Figure 7B). The *J*/*P*_*N*_ ratio measured in ET101-inoculated tomato and rice plants did not show any significant differences between the S0 and S2 treatments. However, a significant increase of 174.32% in tomato and 71.83% in rice compared with respective controls (C0) was observed in leaves of ET101-inoculated plants subjected to S4 stress treatment (Figure 7B).

**FIGURE 8:**
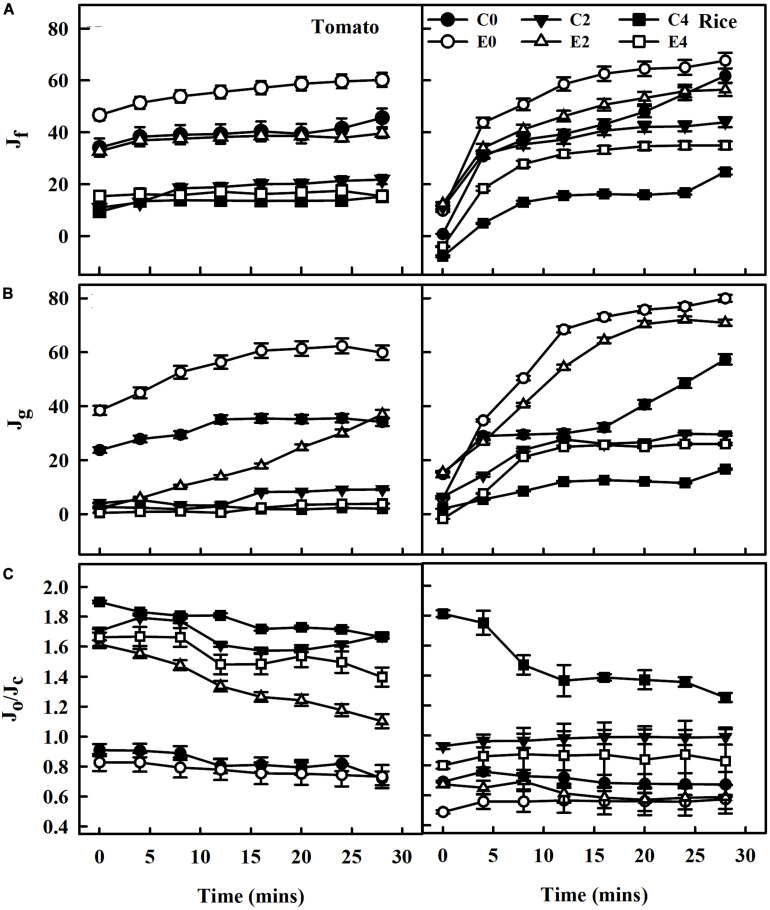
The rate of electron transport derived from gas exchange and chlorophyll fluorescence measurements during photosynthetic induction following dark-adapted state. **(A)** Electron transport rate (*J*_*f*_) calculated based on measurements of PSII quantum yields, assuming the equal distribution of absorbed light between PSI and PSII in leaves of tomato and rice plants subjected to salinity stress at 1,500 μmol photons m^–2^ s^–1^ PPFD up to saturation of 30 min. **(B)** Rate of electron transport consumed by carboxylation plus oxygenation of RuBP (*J*_*g*_), calculated by the data from gas exchange measurements. **(C)** Ratio of electron transport consumed by photorespiration (*J*_*o*_) to the total PSII electron transport (*J*_*c*_) in leaves of tomato and rice plants (*J*_*o*_/*J*_*c*_). The average values ± standard errors from triplicates of sample size *n* = 6 are presented. The open symbols denote ET101-inoculated plants, whereas the closed symbols represent uninoculated tomato and rice plants (C0: control + 0 mM NaCl, C2: control + 200 mM NaCl, C4: control + 400 mM NaCl; E0: ET101 + 0 mM NaCl, E2: ET101 + 200 mM NaCl, E4: ET101 + 400 mM NaCl).

At photosynthetically active radiation of 1,500 μmol m^–2^ s^–1^, the rate of electron transport of PSII (*J*_*f*_) calculated using chlorophyll fluorescence data decreased with increasing salinity stress in both tomato and rice plants. Although the ET101-inoculated tomato and rice plants E0 had higher electron transport (*J*_*f*_), the leaves of the plants exposed to E2 and E4 stress showed drastic decrease in electron transport (*J*_*f*_) but still had higher rates than that of C2 and C4 uninoculated salt-stressed plants (Figure 8A). Compared with the ET101-inoculated tomato and rice plants, the fraction of electron transport consumed by carboxylation plus oxygenation of RuBP (*J*_*g*_) calculated by the data from gas exchange measurements decreased in C0, C2, and C4 plants. While the ETR (*J*_*g*_) to support photosynthesis process followed the trend of PSII ETR (*J*_*f*_), the *J*_*g*_ values of each treatment in both uninoculated and ET101-inoculated plants were substantially lower under salt stress than the *J*_*f*_ (Figure 8B). The higher values of *J*_*f*_ may indicate the presence of alternative electron flow. The leaves of ET101-inoculated plants showed considerable increase in ETR *J*_*f*_ and *J*_*g*_ in the respective treatment controls; however, at stress conditions, the rates are decreased (Figure 8B). The distribution of electrons within linear electron transport between photorespiration and RuBP carboxylation estimated as *J*_*o*_/*J*_*c*_ decreased in S0 and remained higher in S2 and S4 treatment of both plants. The *J*_*o*_/*J*_*c*_ ratio of distribution of electrons between carboxylation and oxygenation indicates the steady state of electron flow in uninoculated (C0) and ET101-inoculated (E0) plants in both the tomato and rice plants even at 30 min of photosynthetic induction at high light. Compared to the ET101-inoculated E2 and E4 plants, the C2- and C4-treated tomato and rice plants exhibited higher electron flow toward oxygenation rather than carboxylation. In severely stressed control plants C4, the proportion of electrons consumed in oxygenation was 130.74% higher in tomato and 86.24% in rice plants (Figure 8C).

**FIGURE 9:**
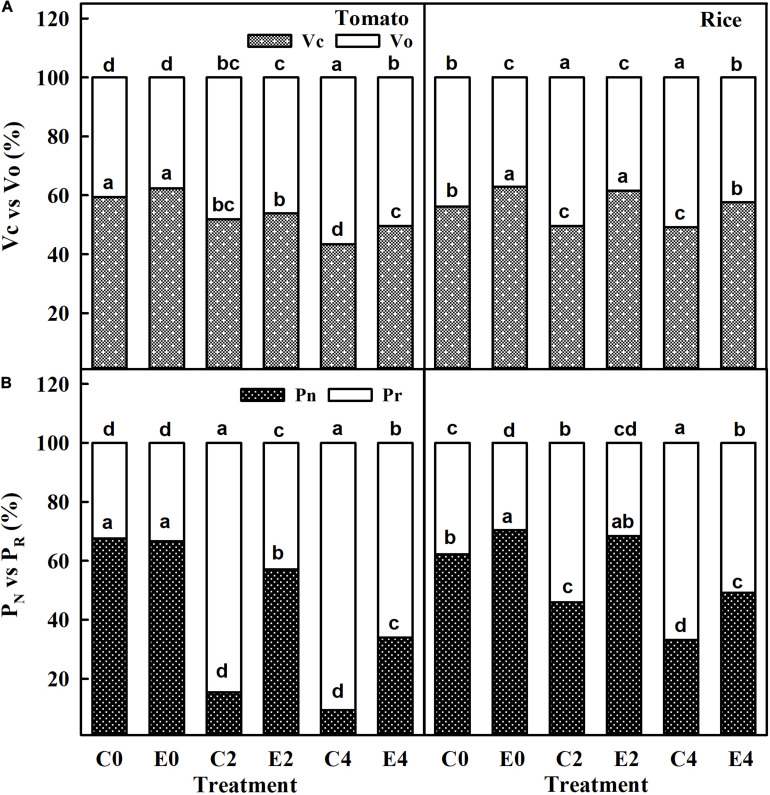
**(A)** Carboxylation (*V*_*C*_) and oxygenation (*V*_*o*_) catalyzing efficiency of RuBisCO enzyme in uninoculated and ET101-inoculated tomato and rice plants subjected to salinity stress conditions. **(B)** Rate of net photosynthesis (*P*_*N*_) and rate of photorespiration (*P*_*R*_) calculated by the data from simultaneous gas exchange and chlorophyll fluorescence measurements in uninoculated and ET101-inoculated tomato and rice plants subjected to salinity stress conditions (C0: control + 0 mM NaCl, C2: control + 200 mM NaCl, C4: control + 400 mM NaCl; E0: ET101 + 0 mM NaCl, E2: ET101 + 200 mM NaCl, E4: ET101 + 400 mM NaCl). Bars represent mean ± SE of triplicates. Different letters on the bars indicate significant difference between the salt treatments and bacterial inoculation (ANOVA; *P* < 0.001). Other details are mentioned in Section “Materials and Methods.”

The carboxylation rate (*V*_*C*_) in uninoculated and ET101-inoculated tomato and rice plants was significantly decreased in C2, C4, E2, and E4 plants in a stress-dependent manner. However, leaves of ET101-inoculated tomato and rice plants showed higher *V*_*C*_ rates in control and stress conditions than leaves of uninoculated plants (Figure 9A). The C4 stress-treated uninoculated tomato and rice plants showed lowest *V*_*C*_ rates with 26.88% decrease in tomato plants and 12.40% decrease in rice plants compared to uninoculated controls at S0. Contrary to the observations with regard to *V*_*C*_, the oxygenation rate (*V*_*o*_) was lower in leaves of ET101-inoculated tomato and rice plants at E0 condition than in uninoculated plants (C0). The *V*_*o*_ rates were maximum in leaves of uninoculated tomato (39.25% increase from C0) and rice plants (15.87% increase from C0) subjected to C4 stress conditions (Figure 9A). Compared to uninoculated plants, leaves of ET101-inoculated tomato plants showed increased *P*_*N*_ rates by 270.82 and 264.14% under S2 and S4 salinity stress conditions and 48.87 and 48.84% in rice plants, respectively (Figure 9B). The *P*_*R*_ rates were significantly lower in E2 and E4 stress conditions in both tomato and rice plants compared to the controls and C2 and C4 plants (Figure 9B). Leaves of uninoculated tomato and rice plants under high salinity stress (C4) showed increased *P*_*R*_ of 179.47% in tomato and 76.87% in rice compared to respective controls. However, in case of ET101-inoculated plants subjected to E4 stress, the *P*_*R*_ rates were 97.82% in tomato and 71.63% in rice plants (Figure 9B).

### Adenylate Levels in Uninoculated and Inoculated Tomato and Rice Plants Exposed to Salinity Stress Conditions

**FIGURE 10:**
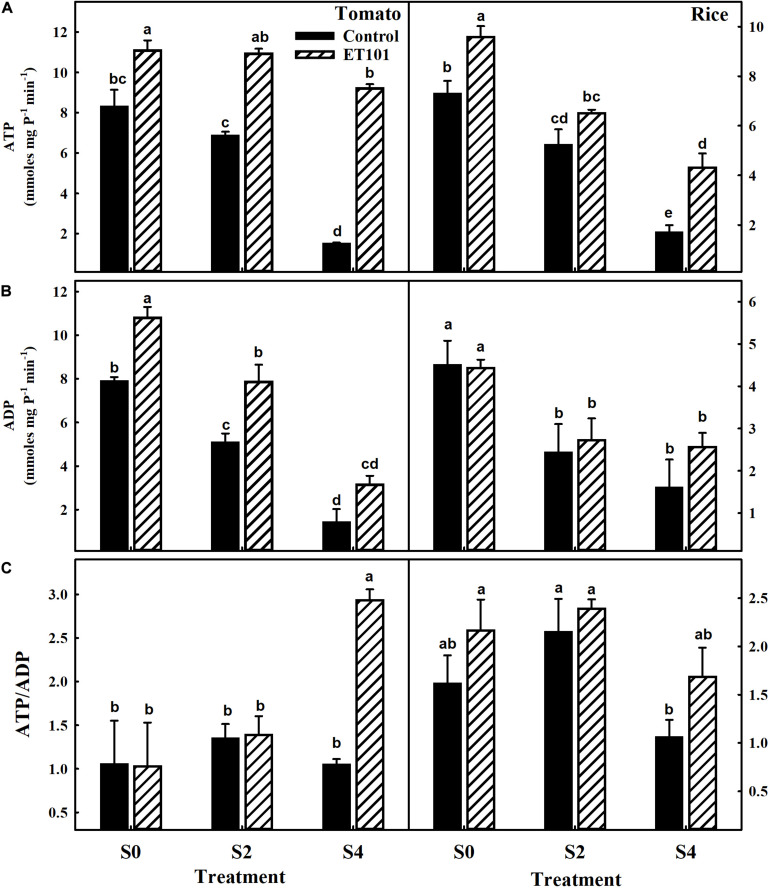
**(A)** Estimation of ATP levels, **(B)** estimation of ADP levels, and **(C)** ratio of ATP to ADP levels in leaves of uninoculated and inoculated tomato and rice plants subjected to different salt stress treatments (C0: control + 0 mM NaCl, C2: control + 200 mM NaCl, C4: control + 400 mM NaCl; E0: ET101 + 0 mM NaCl, E2: ET101 + 200 mM NaCl, E4: ET101 + 400 mM NaCl). Bars represent mean ± SE of triplicates. Different letters indicate significant difference between the salt treatments and bacterial inoculation (ANOVA; *P* < 0.001). Other details are mentioned in Section “Materials and Methods.”

Under salinity stress conditions, the production of ATP and ADP in C2 and C4 plants decreased in both the tomato and rice plants (Figures 10A,B). However, in inoculated tomato and rice plants at E2 and E4 treatments, the ATP levels are either maintained or increased than unstressed uninoculated plants (Figures 10A,B). The drastic changes in levels of adenylates (ATP, ADP, and ATP/ADP) act as proof for disruption of total cellular respiration in plants by the salinity stress. The increase in ATP/ADP levels was observed in leaves of ET101-inoculated tomato and rice plants under control and salt stress conditions (Figure 10C).

### ROS Production in Leaves of Uninoculated and Inoculated Tomato and Rice Plants Exposed to Salinity Stress

**FIGURE 11:**
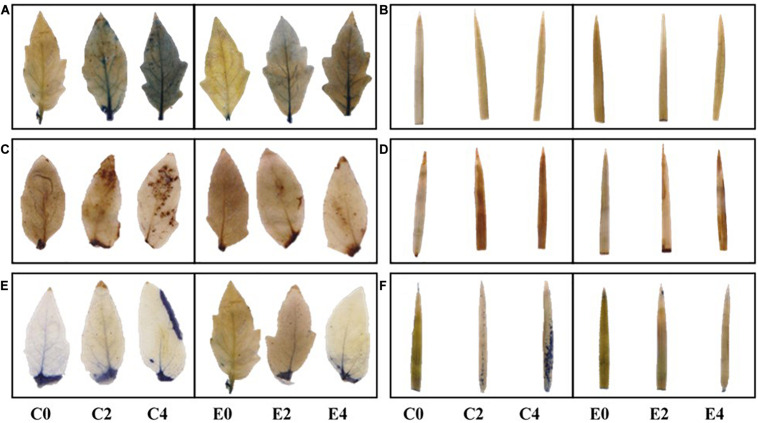
Changes in ROS levels and cell death assay in leaves of uninoculated and inoculated tomato and rice plants. **(A,B)** *In vivo* superoxide ion O${}_{2}^{-}$ localization in uninoculated and inoculated leaves of tomato and rice plants subjected to different salt stress conditions using NBT (nitroblue tetrazolium) staining. **(C,D)** *In vivo* H_2_O_2_ localization in uninoculated and inoculated leaves of tomato and rice plants treated with different salt stress conditions using DAB (diaminobenzidine) staining. **(E,F)** *In vivo* localization of dead cells in uninoculated and inoculated leaves of tomato and rice plants subjected to different salt stress conditions using trypan blue staining. Other details are mentioned in Section “Materials and Methods.”

The salinity stress in plants leads to the accumulation of ROS, which can cause cellular damage leading to cell death. The ROS produced in leaves of the plants subjected to salinity stress can be detected by histochemical staining. The accumulation of H_2_O_2_ and superoxide ions in the leaf tissues of inoculated and uninoculated tomato and rice plants has been visualized by DAB and NBT staining. The uninoculated plants grown under salinity stress conditions showed an increase in H_2_O_2_ and superoxide levels in leaves. However, leaves of plants inoculated with ET101 isolate showed considerable decrease in both H_2_O_2_ and superoxide levels under E2 and E4 salinity stress conditions (Figures 11A–D). The substantial decrease in ROS accumulation in ET101-treated plants under salinity stress emphasizes the importance of the *S. sciuri* in ameliorating the ROS levels inside the plant cells. Trypan blue is a vital stain typically used for visualization of dead cells in tissues. While intense blue staining is observed in leaves of uninoculated tomato plants subjected to S4 salinity stress, the blue spots were marginal in leaves of ET101-inoculated tomato plants subjected to S4 salinity stress indicating the protection afforded by ET101 isolate against salinity stress induced cell death (Figures 11E,F).

## Discussion

### *S. sciuri* Is a Halophilic PGPR

In the current study, we have isolated a halotolerant bacterium, *S. sciuri* ET101, which has the potential to improve plant growth under salinity stress conditions. Rhizospheric halotolerant bacteria with plant growth promotion ability are known to be the prime candidates to test their capability to alleviate salt stress symptoms in crop species (Akram et al., 2016; Bharti et al., 2016; Ilangumaran and Smith, 2017; Shahid et al., 2019; Bakka and Challabathula, 2020; Kumar et al., 2020). The isolation and employment of potent rhizospheric halotolerant bacteria were shown to have significant beneficial effect on plant growth and development under varied abiotic stress conditions (Palacio-Rodríguez et al., 2017; Sapre et al., 2018; Vives-Peris et al., 2018; Prittesh et al., 2020). Although many species of *Staphylococcus* genus are commensal pathogens of animals and plants (Prithiviraj et al., 2005; Nemeghaire et al., 2014), the species of *Staphylococcus* possessing various plant growth-promoting traits have also been reported (Khan et al., 2015). Salt-stressed maize plants inoculated with halophilic *S. sciuri* SAT-17 isolate showed plant growth-promoting capability along with increased salinity tolerance and enhanced antioxidative defense (Akram et al., 2016). Maize plants inoculated with the bacterial strains (STN-1 and STN-5) taxonomically classified as *Staphylococcus* spp. showed enhanced growth along with increased antioxidant enzyme activity and decreased ROS under salt stress conditions (Shahid et al., 2019). Production of plant hormones and plant beneficial compounds are key factors for considering a bacterium as PGPR. As IAA acts as a major contributor for enhancement of plant growth by root elongation (Liu et al., 2018), the bacterial production of IAA by tryptophan-independent and tryptophan-dependent means and the production of GA confirm the bacteria to be a PGPR. Additionally, the production of ACC-deaminase activity in the presence of NaCl in the culture medium is evident by the production of α-ketobutyrate. Possessing the ACC deaminase activity by bacteria is an important trait for rendering stress tolerance to the plants as it reduces the production of ACC by cleaving it into α-ketobutyrate and ammonia (Glick, 2014). Although the isolate is a non-nitrogen fixer, it has the ability to produce ammonia from nitrogen-containing organic matter. Similar to our results, the inoculation of exopolysaccharide and ACC-deaminase producing *Bacillus* spp. showed higher germination rate and increased salinity tolerance in wheat seedlings under *in vitro* conditions (Amna et al., 2019). Results suggest that the traits possessed by ET101 isolate convincingly confirm it as halophilic PGPR.

### *S. sciuri* Protected Tomato and Rice Plants From Salinity Injury

Plants experiencing salinity stress downregulate and shut down photosynthesis mainly due to stomatal closure (Chaves et al., 2009; Igamberdiev, 2015). The leaf RWC serves as a principal factor for monitoring the water status in plants. Several studies have reported less decrease in RWC exhibited by tolerant genotypes than the sensitive plants due to salinity stress (Mekawy et al., 2015; Hossain and Dietz, 2016; Negrão et al., 2017). Salinity stress induces the osmotic stress in plants causing the depletion of water content in leaves with devastating effect on the cellular membranes and organelles. Compared to uninoculated plants, the lesser EL observed in the ET101-inoculated plants could possibly be due to the production of osmolytes and compatible solutes required for cellular protection. The proline and glycine betaine are the most important and efficient compatible solutes accumulated in many of the living organisms during stress conditions (Slama et al., 2015). Proline acts as a molecular chaperone to protect the biological macromolecules during abiotic stress conditions, and glycine betaine is an osmoprotectant known to protect PSII during salinity. Additionally, the integrity of the membranes and the activity of the key enzymes are regulated by the accumulated glycine betaine during stress conditions (Chen and Murata, 2008; Giri, 2011). Exogenous application of proline and/or glycine betaine to salt-stressed rice plants improved plant growth and salinity tolerance suggesting the importance of osmolytes for abiotic stress tolerance (Wutipraditkul et al., 2015). The higher accumulation of proline and glycine betaine in ET101-inoculated tomato and rice plants are important for cellular protection during salt stress conditions. Our results are in congruence with reports on salinity tolerance in various plants by the inoculation of PGPR (Singh et al., 2015; Khan et al., 2016; Cordero et al., 2018; Sapre et al., 2018; Sarkar et al., 2018; Prittesh et al., 2020).

Inoculated plants performed better than the uninoculated plants by showing higher plant growth rate, higher RWC, and maintenance of cellular integrity as indicated by EL. All these parameters serve as good indicators of salt tolerance in glycophytes (Neto et al., 2014; Kumar et al., 2020). The development of root system and architecture is important for the plants to acquire water and nutrition from the soil and thereby increase the replacement rate of water lost during transpiration. Higher proliferation of roots was observed in ET101-inoculated plants with increased lateral root formation and growth (data not shown), thereby allowing better penetration into the soil to acquire water and nutrition. Salt stress is known to cause damage to the chloroplast structure and instability of the chlorophyll protein complexes resulting in decreased chlorophyll content of leaves (Parida and Das, 2015; Zhang et al., 2020). Although this is true for uninoculated tomato and rice plants, the inoculated plants accumulated greater amounts of photosynthetic pigments (chlorophyll and carotenoids). The ability of plants to synthesize more chlorophyll during salinity stress is a perceptible criterion to a stress-tolerant species. Tolerant genotypes of rice cultivars contained significantly higher chlorophyll content than sensitive genotypes under salinity conditions (Kanawapee et al., 2012). Because higher chlorophyll content contributes to the improvement of photosynthesis process in plants (Pavlović et al., 2014), the PGPR-inoculated plants performed better in terms of photosynthesis than the uninoculated plants. In comparison to these, ET101-inoculated plants showed higher chlorophyll and tend to gain more stress tolerance than the uninoculated plants. Many reports are published showing increased chlorophyll content in plants with PGPR inoculation (Li and Jiang, 2017; Ansari et al., 2019; Prittesh et al., 2020). The presence of high amount of photosynthetic pigments in the PGPR-inoculated plants aids in increasing the biochemical rate of CO_2_ fixation, thereby maximizing the net photosynthesis and growth rate of plants during normal and salt stress conditions.

### *S. sciuri* Modulates Photosynthetic Responses in Tomato and Rice Plants Subjected to Salinity Stress

A decline in net photosynthesis rate during salinity stress is due to lower intracellular CO_2_ levels due to stomatal closure. The *P*_*N*_/Ci curves showed the sensitivity of the photosynthesis to salt stress conditions and the induction in photosynthesis to increasing CO_2_ levels in leaves of both uninoculated and inoculated tomato and rice plants. However, the induction is more in leaves of inoculated tomato and rice plants. Photosynthetic CO_2_ fixation is regulated by both stomatal and non-stomatal limitations. The reduction in *P*_*N*_ in leaves under salinity stress is associated with the increase in intercellular CO_2_ (Ci) content in both tomato and rice plants. Stomatal closure during salinity stress is an adaptive measure employed by the plants to minimize water loss during transpiration (Engineer et al., 2016). In our study, the decrease in *P*_*N*_ and *g*_*s*_ is observed in tomato and rice plants subjected to salt stress treatment. Higher *P*_*N*_ along with high *g*_*s*_ rates was observed in tolerant variety IR651 during salinity stress (Foad and Abdelbagi, 2007). As increased photosynthetic capacity is directly linked with the increase in yields (Ambavaram et al., 2014), the employment of bacteria for protecting the photosynthesis during salinity stress and amelioration of salt stress symptoms in plants by bacteria can be considered a feasible approach. Reduction in *g*_*s*_ and transpiration (*E*) rate are also important adaptive mechanisms of plants for salinity tolerance (Flowers and Yeo, 1981). Impairment of ATP synthesis could also be one of the reasons apart from stomatal closure for decrease in photosynthesis during salinity stress (Cruz et al., 2004). The chloroplasts have the capacity to cope up with the changes for energy demands under stress conditions (Kramer and Evans, 2010). The optimization of ATP and NADPH ratio generated during the light-dependent reactions plays a major role in the prevention of over reduction of chloroplast electron transport chain and ROS production. In our study, although ET101-inoculated plants showed stable ATP/ADP ratio during S0 and S2 stress conditions, the ratio increased significantly during S4 stress conditions due to substantial decrease in ADP levels in tomato plants (Figure 10). Decreased ADP levels in S4 plants can possibly be due to suppressed production of ADP or higher phosphorylation of ATP.

Increased photosynthesis during salinity stress conditions can be due to increase in stomatal conductance, possessing a large number of open stomata, or due to high photosynthetic pigment content. Salinity impacted the photosynthesis of both tomato and rice plants by reducing stomatal conductance and leading to decreased diffusion of CO_2_ to the carboxylation sites. Salinity stress negatively affects the RuBisCO activity by modulating its rate of biosynthesis and degradation. The Ci/Ca ratio indicates the non-stomatal limitation, which is controlled by enzymatic CO_2_ fixation of RuBisCO and production of carbohydrates in the Calvin cycle (Ueda et al., 2013). The observed increase of Ci/Ca ratio in S2- and S4-treated uninoculated plants depicts the non-stomatal limitations in usage of CO_2_ by RuBisCO enzyme. The Stevia plants inoculated with the plant growth-promoting *Streptomyces* spp. promoted the accumulation of the RuBisCO protein along with the maintenance of its stability under salinity stress conditions (Tolba et al., 2019). The ET101-inoculated plants sustained the level of Ci/Ca ratio indicating the maintenance of balance between carboxylation and oxygenation of RuBisCO enzyme by enhancing the availability of CO_2_. The plants inoculated with ET101 isolate showed an increase in photosynthesis along with stomatal conductance, suggesting bacterial-mediated increase in photosynthesis in plants.

Chlorophyll fluorescence analysis serves as an essential and quick tool for evaluation of plant survival and performance in response to salinity stress (Baker and Rosenqvist, 2004). Salinity stress downregulates photosynthesis and diminishes the quantum yield and efficiency of PSII. In our study, uninoculated plants exposed to S2 and S4 salinity treatments exhibited strong inhibition of photosynthetic capacity, along with diminished PSII activity. As the light-dependent reactions are disrupted during salinity stress, the excess reducing equivalents should be dissipated via non-photochemical processes such as heat or chlorophyll fluorescence to avoid damage to the leaf tissues (Genty et al., 1989; Maxwell and Johnson, 2000). Decreased *F*_*v*_/*F*_*m*_ indicates that PSII reaction center is damaged subsequent to photoinhibition (Murata et al., 2007). In this study, *F*_*v*_/*F*_*m*_ was affected when plants were subjected to salt stress indicating damage to the PSII reaction center. However, ET101-inoculated tomato and rice plants showed marginal decrease in *F*_*v*_/*F*_*m*_ indicating that quantum efficiency of PSII is well maintained by the bacterial inoculation. The actual quantum yield of PSII photochemistry (ΦPSII) was also enhanced by ET101 inoculation. The increase in the NPQ is the major process carried out by plants to prevent photo damage induced by salinity stress (Maxwell and Johnson, 2000; Stepien and Johnson, 2009). Apparently, the increase in NPQ in ET101-inoculated plants was effective in preventing the decline of *F*_*v*_/*F*_*m*_. The recovery of chlorophyll fluorescence parameters upon inoculation of plants with ET101 confirms the tolerance of the plants to salt stress conditions. The dissipation of excitation energy by photochemical utilization contributes to downregulation of PSII to avoid overreduction of primary electron acceptor Q_*A*_ (Zribi et al., 2009). The study of Wu et al. (2018) stated that the reduction in ΦPSII and increase in NPQ are the adaptive responses of salt stress in cucumber plants. In our study, ΦPSII and *qP* were significantly declined by salt stress, whereas NPQ is increased in both uninoculated and inoculated plants during salt stress. The shutdown of active photosystem II reflects in higher NPQ due to reduction in the quantity of quantum light absorbed by the reaction centers of photosystem II (Wu et al., 2018). Although the E4 plants exhibited higher NPQ rates, the increase in ΦPSII confirms the active photosystem II activity and importance of bacteria for rendering the protection to photosystem II to tomato and rice plants experiencing salinity stress. Although both uninoculated and inoculated plants showed damage to PSII during salinity stress, our results showed a dramatic decrease in the reduction of photosynthesis in inoculated plants suggesting the positive effect of bacterial inoculation in mitigating salinity stress. The capability of the photosynthetic apparatus to retain its activity ensuring the maintenance of plant productivity is associated with the activation of photo protective mechanisms during bacterial inoculation.

Salinity stress causes imbalance between PSII photochemistry and electrons required for efficient photosynthesis leading to photo inhibition culminating in a stronger decrease in, apparent RuBisCO efficiency (*P*_*N*_/Ci) (Pérez-López et al., 2013; Wang et al., 2017). The partitioning of reductive power between photosynthesis and alternative sinks, which consume electrons, could be estimated by *J*/*P*_*N*_ ratio. The higher *J*/*P*_*N*_ ratio exhibited in tomato and rice plants during S4 stress treatment indicates the downregulation CO_2_ assimilation mechanism by salinity stress and the divergence of the flow of electron transport to other metabolic processes. Conversely, in ET101-inoculated plants, the *J*/*P*_*N*_ ratio was lowered compared to uninoculated plants, indicating involvement of bacterial-derived processes, which drive electron flow to carbon metabolism, reflecting enhanced photosynthesis rate.

### Decreased ROS in *S. sciuri* Inoculated Tomato and Rice Plants Is Important for Cellular Homeostasis

The reduction in carboxylation activity of RuBisCO is correlated with the decrease in *P*_*N*_/Ci ratio and excessive generation of ROS. The oxidative damage occurs due to the imbalance between ROS production and quenching. Strong NBT and DAB staining was observed in C4 plants in both tomato and rice plants, indicating that they have higher stress levels than E2 and E4 plants. The NBT and DAB staining of leaves from tomato and rice plants indicated that H_2_O_2_ and O${}_{2}^{-}$ anions accumulated in the cells, suggesting that leaf cells under salinity stress are under oxidative stress. Furthermore, the intense staining of trypan blue in C2 and C4 depicts the damages of leaf tissues in leaves of uninoculated plants. H_2_O_2_ and O${}_{2}^{-}$ are primarily generated by the electron transport chain of mitochondria and the membrane-bound PSI electron acceptor found in chloroplast thylakoids (Gill and Tuteja, 2010). The ROS levels were lower in the leaves of inoculated plants than the uninoculated tomato and rice plants, suggesting the likely role of ET101 isolate in activating the plant antioxidant defense mechanism to scavenge the ROS during salinity stress conditions, thereby maintaining cellular redox homeostasis.

### *S. sciuri* Mediated Interplay Between Carboxylation and Oxygenation for Salt Stress Mitigation

The drastic reduction in apparent efficiency of RuBisCO in the uninoculated plants exposed to salinity stress (C2 and C4) suggests the degradation or deactivation of RuBisCO. This difference depicts the imbalance between carboxylase/oxygenase activity, as confirmed by the significant decrease in *V*_*C*_ and increase in *V*_*o*_. The reduction in the synthesis of ATP and NADPH in the uninoculated plants during salinity stress is related to the unavailability of sources needed for CO_2_ fixation in Calvin-Benson cycle (Farquhar and Sharkey, 1982). Photorespiration plays a major role in protecting the plant cells against various abiotic stresses by maintaining the electron flow and activity of Calvin cycle enzymes, thereby preventing the accumulation of enzyme inhibiting metabolites and photoinhibition (Wingler et al., 2000; Voss et al., 2013). Although photorespiration diminishes the potential photosynthetic activity of the plants through catalyzing the oxygenase reaction by RuBisCO, it prevents the damage to the photosynthetic apparatus caused by excessive salinity stress (Salazar-Parra et al., 2015). Adopting combined measurement of gas exchange and Chl fluorescence to calculate *J*_*o*_ and photorespiratory rate, we observed that while the *J*_*o*_ of the uninoculated plants exposed to salinity stress increased (i.e., the photorespiration was activated by the stimulation from salinity stress), the *J*_*o*_ of the inoculated plants decreased significantly. Decrease in the intracellular CO_2_ concentration due to decrease in *g*_*s*_ along with increase in oxygenase activity of RuBisCO enzyme could be the responsible factors for the increase in photorespiratory activity during salinity stress (Farquhar and Sharkey, 1982; Hartman and Harpel, 1994). Correspondingly, *S. sciuri* ET101-inoculated plants sustained relatively high *F*_*v*_/*F*_*m*_ and ΦPSII levels and higher *P*_*N*_ rates along with lower photorespiratory rates. The photorespiratory rates of uninoculated plants increased markedly during salinity stress, and so did *F*_*v*_/*F*_*m*_ and ΦPSII to a relatively low degree. The ET101-inoculated plants during salinity stress maintained a lower photorespiration, thereby providing photoprotection to the stressed plants. The sustainability of relatively high *F*_*v*_/*F*_*m*_ and ΦPSII by ET101-inoculated plants could possibly provide higher photosynthetic capacity. The involvement of photoprotective mechanism as an important contributor for salt tolerance in *Ricinus communis* was shown by Neto et al. (2014).

## Conclusion and Future Perspectives

The results from the current study indicated that the isolated halotolerant bacteria *S. sciuri* ET101 plays a crucial role in protecting the tomato and rice plants against damaging effects of salt stress. The inoculation of bacteria led to a biomass enhancement, increased photosynthetic performance, alterations in leaf gas exchange, and photosynthetic pigment contents under salt stress. The alteration in the apparent RuBisCO activity by the inoculation of *S. sciuri* ET101 in tomato and rice plants can provide novel insights in the plant–microbe interaction mechanisms during salinity stress. The current study paves a way for exploration of photorespiration process in bacterial-mediated alleviation of the salt stress responses in plants. Additionally, the changes in photosynthesis and photorespiration in plants inoculated with halotolerant bacteria need to be explored at molecular level. Furthermore, it is essential to isolate more potent bacteria with PGP traits and gain deeper insights into the bacterial-mediated salinity stress mitigating mechanisms. This will be important for obtaining increased crop growth and higher productivity under salinity stress conditions.

## Data Availability Statement

The raw data supporting the conclusions of this article will be made available by the authors, without undue reservation.

## Author Contributions

ZT and DC designed the research and wrote the manuscript. ZT performed the experiments and interpreted the data. Both authors contributed to the article and approved the submitted version.

## Conflict of Interest

The authors declare that the research was conducted in the absence of any commercial or financial relationships that could be construed as a potential conflict of interest.
