# Supplementary material for: Protection of Photosynthesis by Halotolerant Staphylococcus sciuri ET101 in Tomato (Lycoperiscon esculentum) and Rice (Oryza sativa) Plants During Salinity Stress: Possible Interplay Between Carboxylation and Oxygenation in Stress Mitigation
Source: Front Microbiol. 2021 Jan 8;11:547750. doi: 10.3389/fmicb.2020.547750 (PMC7820118; doi:10.3389/fmicb.2020.547750)
Supplement: Supplementary file 1 [file Data_Sheet_1.docx]

**Frontiers in Microbiology**

**Protection of Photosynthesis by Halotolerant *Staphylococcus sciuri* ET101 in Tomato *(Lycoperiscon esculentum)* and Rice *(Oryza sativa)* plants during Salinity Stress: Possible Interplay between Carboxylation and Oxygenation in Stress Mitigation**

***Zarin Taj Z* and *Dinakar Challabathula****

*Department of Life Sciences, School of Life Sciences, Central University of TamilNadu,*

*Thiruvarur, India – 610 005*

**Supplementary Information**

**Supplementary Figure Legends**

**Supplementary Figure S1**

Phylogenetic tree of *S. sciuri* ET101 strain. The molecular phylogenetic tree of ET101 strain was generated by the neighbour joining (NJ) method using the MEGA 7 program based on the alignment of the DNA sequences from 13 related species. The numbers indicate the frequency with which this node is recovered per 100 bootstrap replications in a total of 1000.

**Supplementary Figure S2.** The effect of *S.sciuri* ET101 on plant growth promotion and increased salt tolerance in tomato and rice plants. Evaluation of plants were made after of salt treatment. (C0 – Control + 0mM NaCl, C2 – Control + 200mM NaCl, C4 – Control + 400mM NaCl; E0 – ET101 + 0mM NaCl, E2 – ET101 + 200mM NaCl, E4 – ET101 + 400mM NaCl). (A) Uninoculated and ET101 inoculated tomato plants; (B) Uninoculated and ET101 inoculated rice plants. Uprooted plantlets were displayed. Other details are mentioned in materials and methods.

**Supplementary Figure S3.** The effect of *S. sciuri* ET101 on plant growth promotion and increased salt tolerance in tomato and rice plants. Evaluation of plants were made after of salt treatment. (C0 – Control + 0mM NaCl, C2 – Control + 200mM NaCl, C4 – Control + 400mM NaCl; E0 – ET101 + 0mM NaCl, E2 – ET101 + 200mM NaCl, E4 – ET101 + 400mM NaCl). (A) Uninoculated and ET101 inoculated tomato plants; (B) Uninoculated and ET101 inoculated rice plants. Other details are mentioned in materials and methods.

**Supplementary Figure S4.** **P_N_ – Ci curve**. The effect of *S.sciuri* ET101 on net photosynthetic rate (P_N_) owing to different CO_2_ ranges from 40 to 1000 µmol CO_2_ mol^-1^_._ ). (A) Uninoculated and ET101 inoculated tomato plants; (B) Uninoculated and ET101 inoculated rice plants. Values represent mean of triplicates from sample size n=6. The open symbols denotes ET101 inoculated plants whereas the closed symbols represents uninoculated tomato and rice plants. (C0 – Control + 0mM NaCl, C2 – Control + 200mM NaCl, C4 – Control + 400mM NaCl; E0 – ET101 + 0mM NaCl, E2 – ET101 + 200mM NaCl, E4 – ET101 + 400mM NaCl).

**Supplementary Figure S5.** The effect of *S. sciuri* ET101 on plant growth promotion and increased salt tolerance in tomato and rice plants. Evaluation of plants were made after salt stress treatment. (C0 – Control + 0mM NaCl, C2 – Control + 200mM NaCl, C4 – Control + 400mM NaCl; E0 – ET101 + 0mM NaCl, E2 – ET101 + 200mM NaCl, E4 – ET101 + 400mM NaCl). (A) Shoot length; (B) Root length; and (C) Biomass of uninoculated and ET101 inoculated tomato and rice plants. Bars represents mean ± SE of triplicates. Different letters indicate significant difference between the salt treatments and bacterial inoculation (ANOVA; P= <0.001).Other details are mentioned in materials and methods.

**Supplementary Figure S6:**

Root Colonization of *S. sciuri* ET101 in tomato and rice plants. (A) Colonies of *S. sciuri* ET101 obtained from the inoculated soil; (B) Colonies of *S. sciuri* ET101 obtained from the outer surface of plant roots; and (C) Colonies of *S. sciuri* ET101 obtained from inside plant root tissues.

**Supplementary Figure S1**

**Supplementary Figure S2**


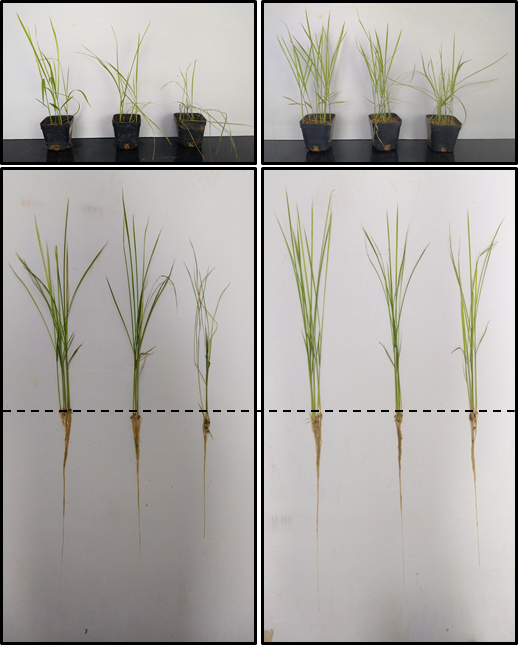

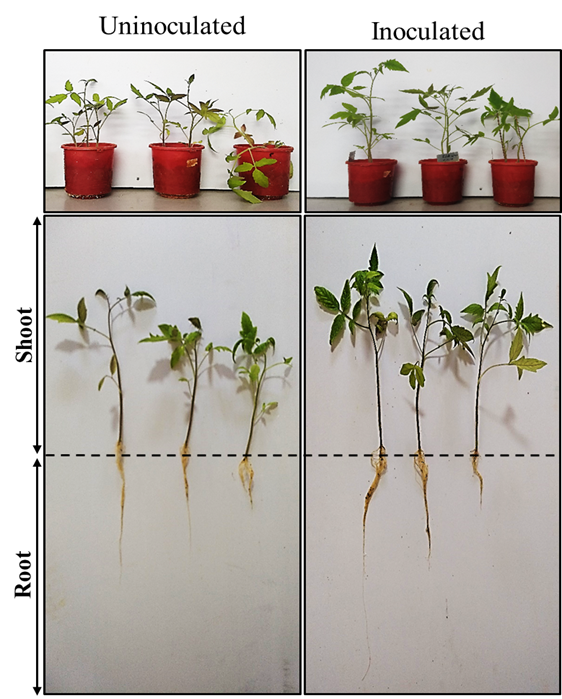


**C0 C2 C4**

**C0 C2 C4**

**E0 E2 E4**

**E0 E2 E4**

**A**

**B**

**Supplementary Figure S3**


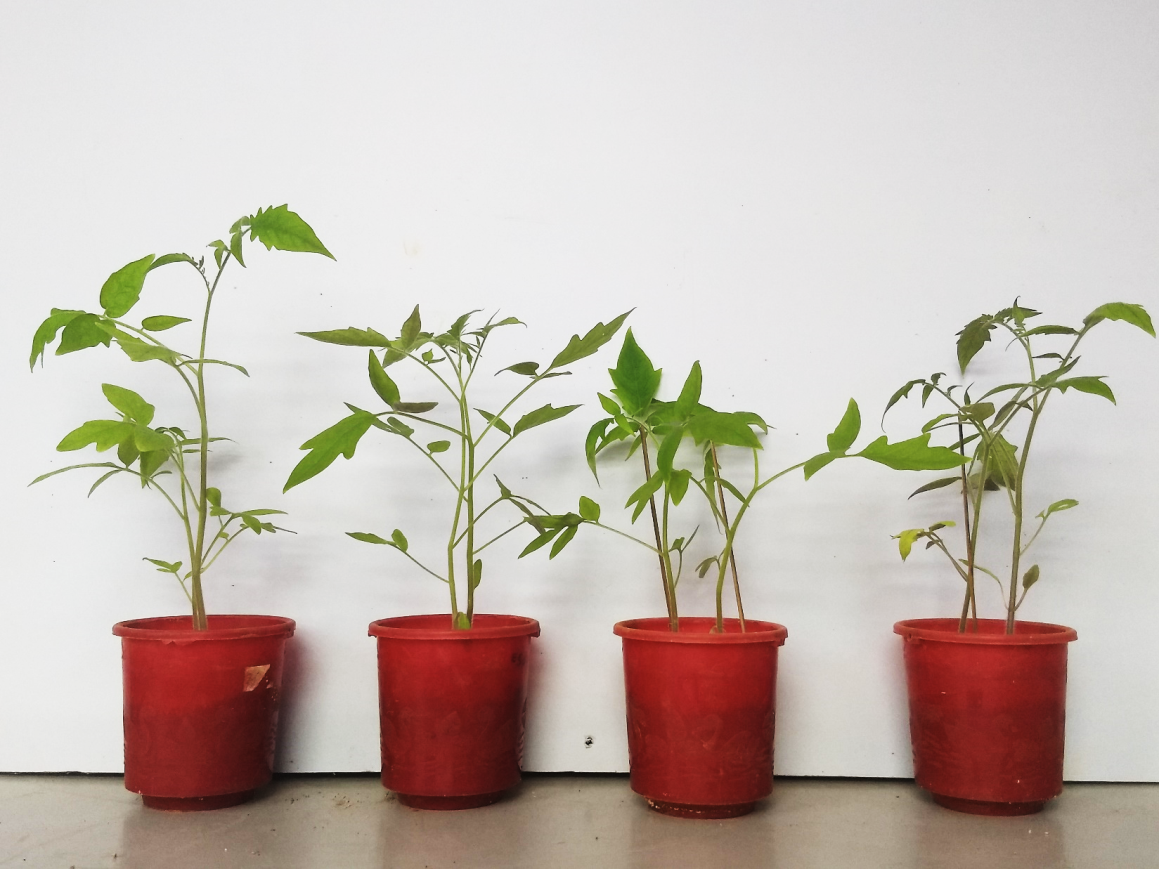

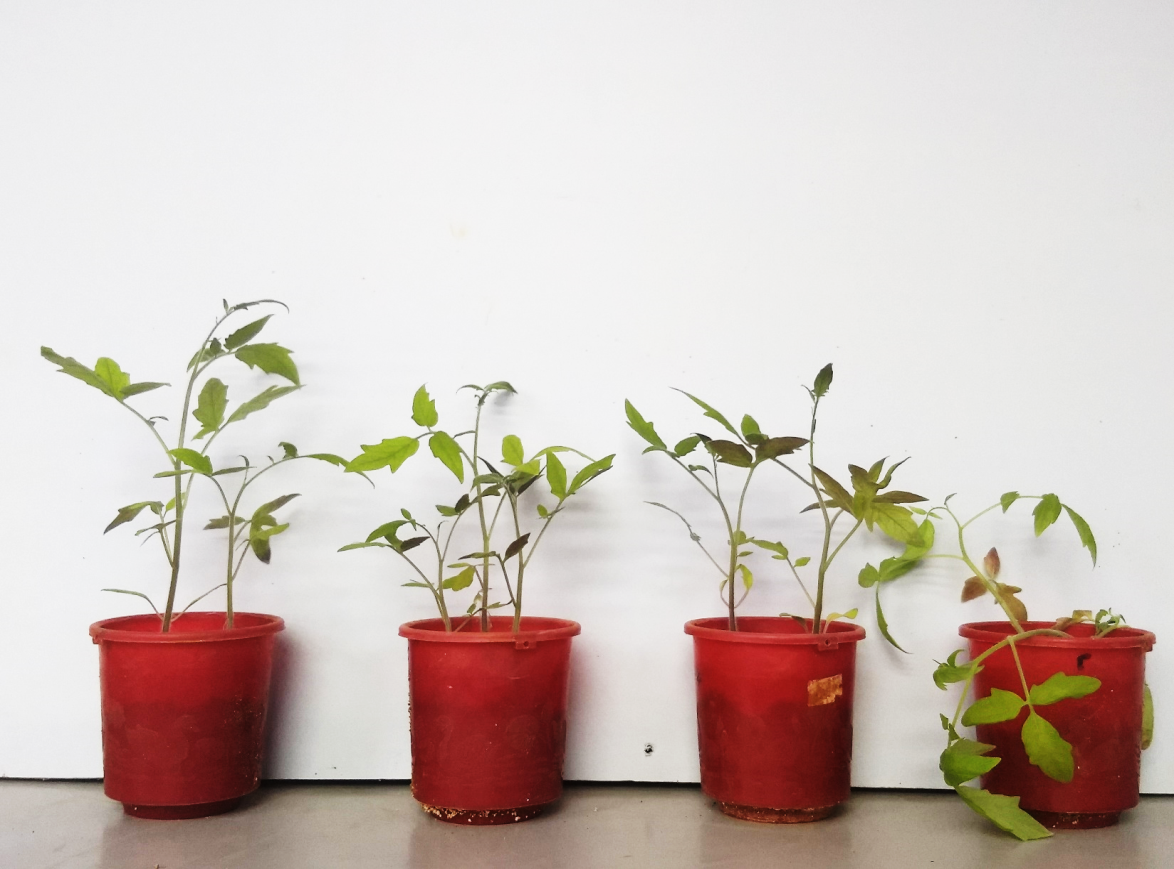


**E0**

**E2**

**E4**

**C2**

**C0**

**C4**

**A**


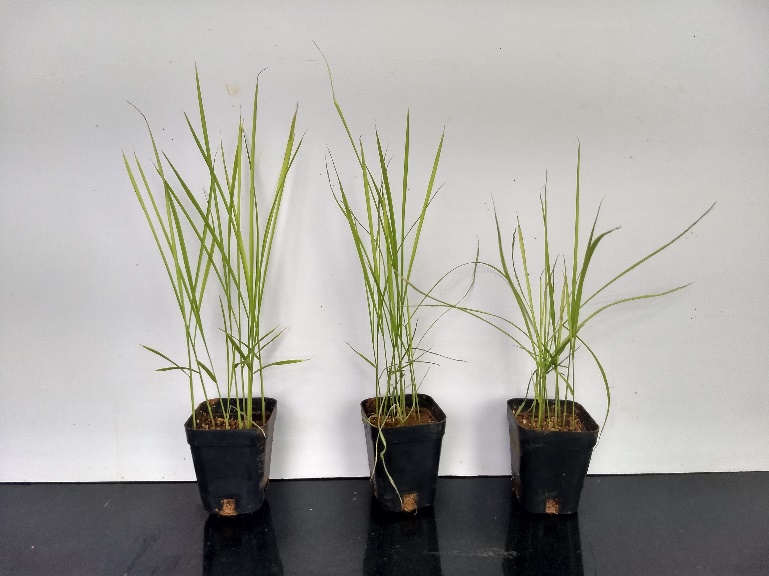


**E0**

**E2**

**E4**


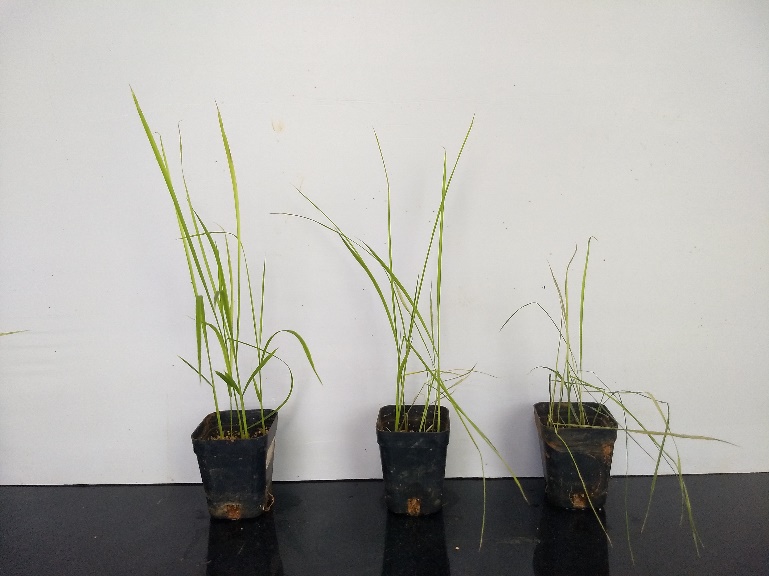


**C2**

**C0**

**C4**

**B**

**Supplementary Figure S4**


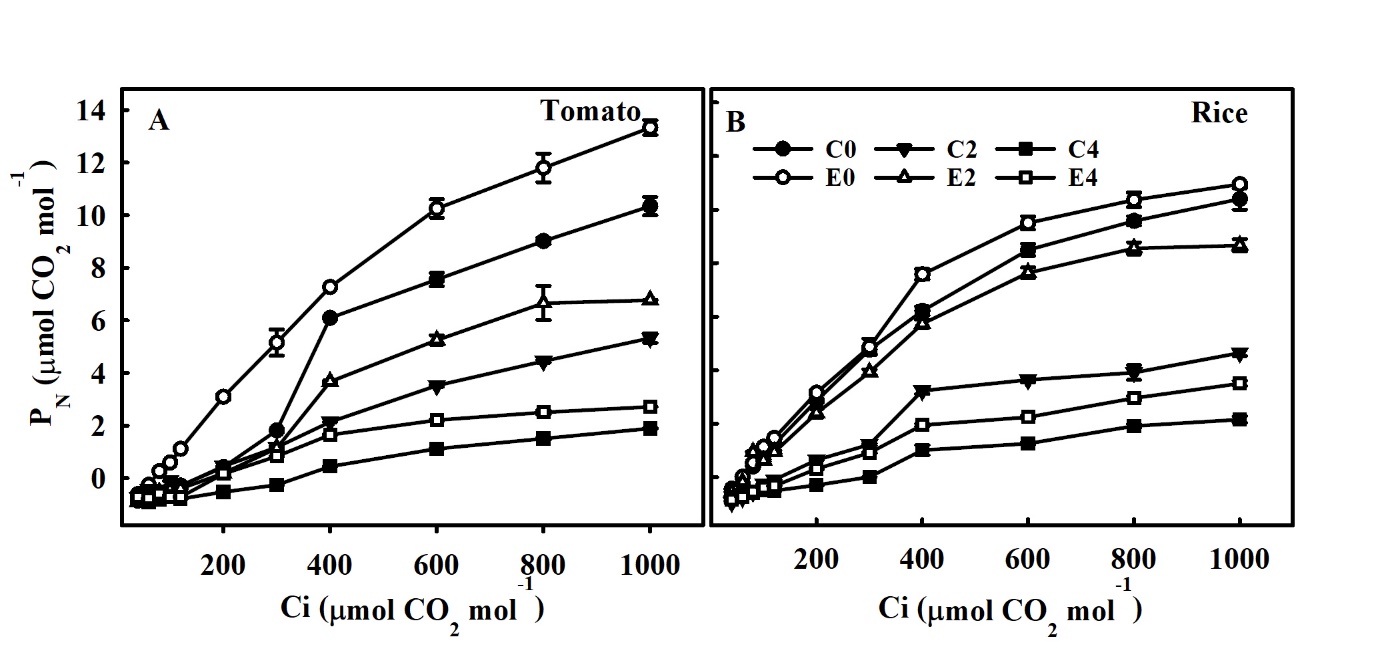


**Supplementary Figure S5**

**
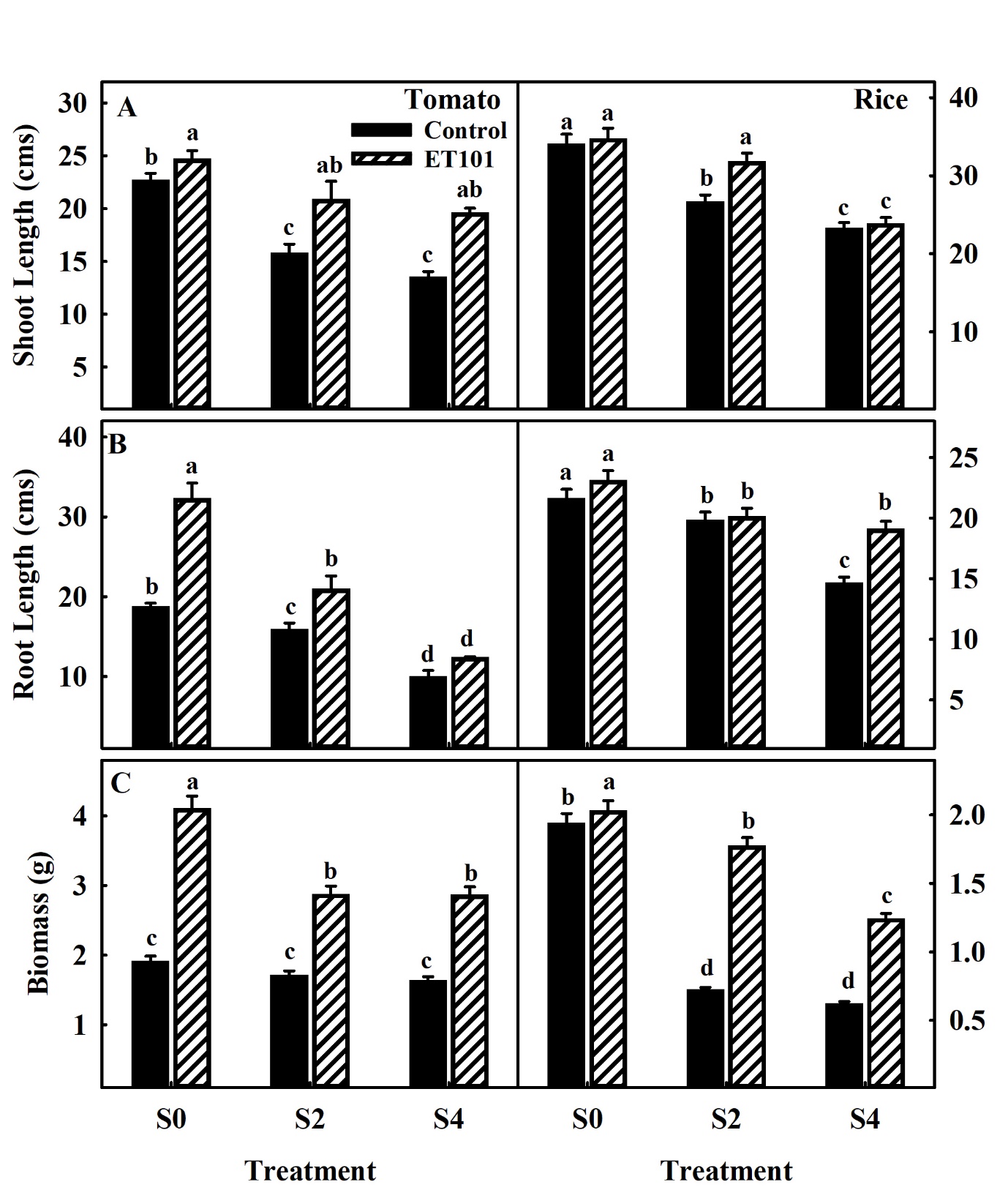
**

**Supplementary Figure S6**


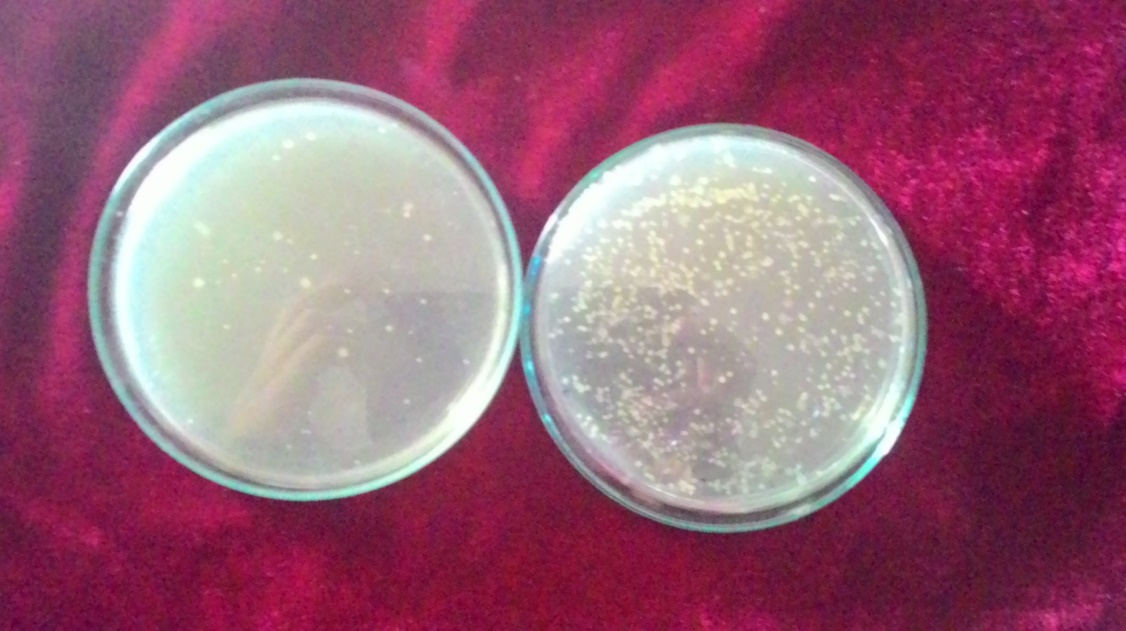

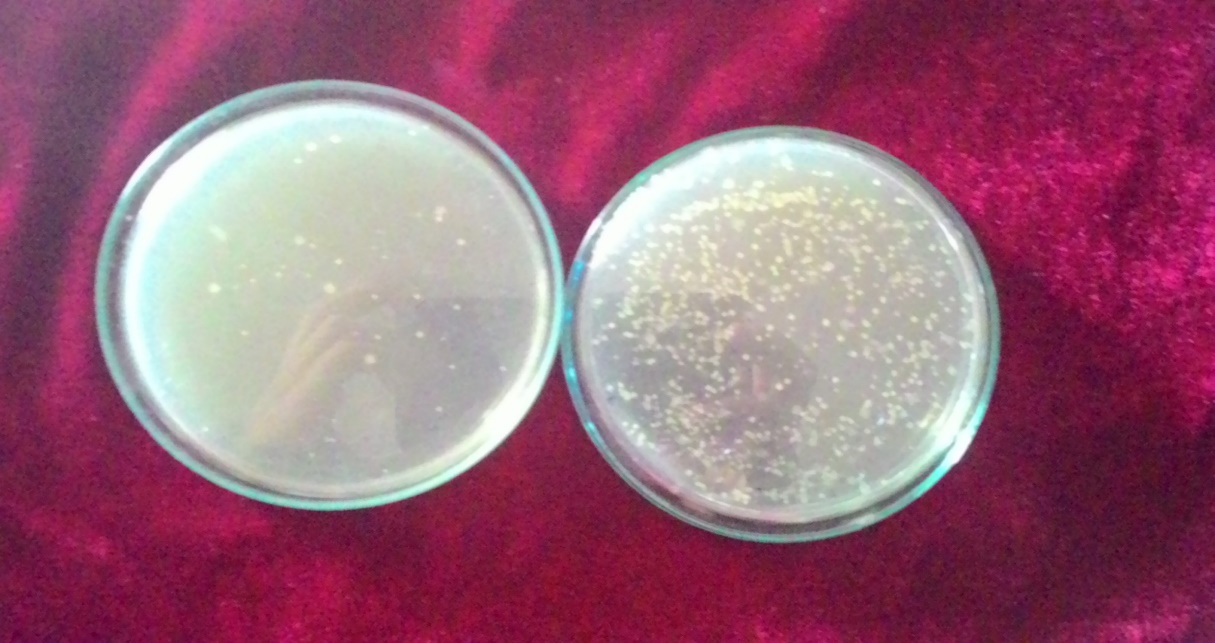


**A**


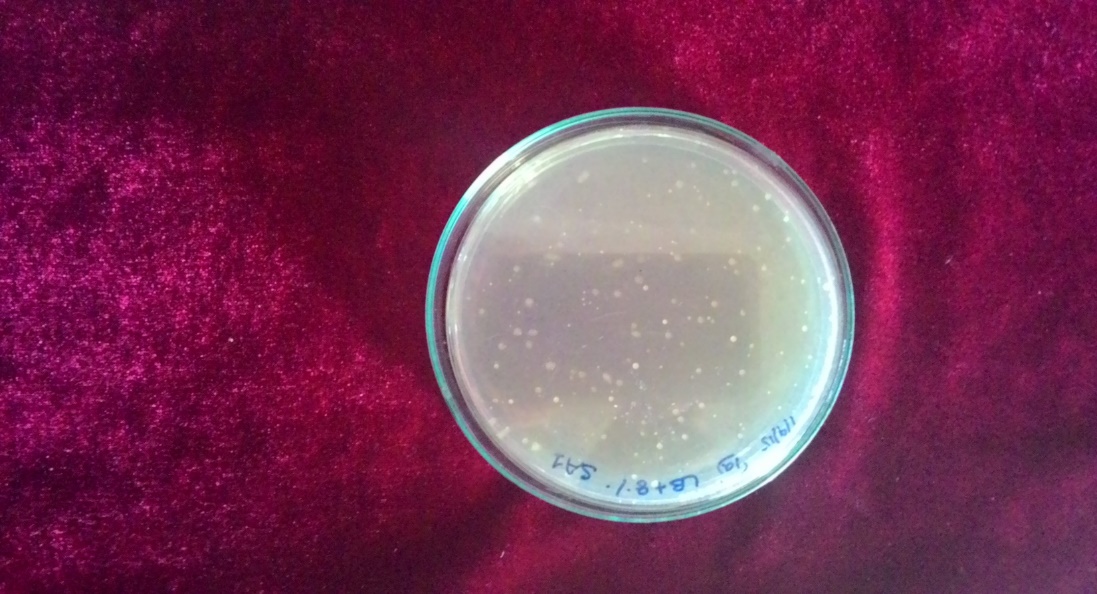


**B**

**C**

| **Sources** | **CFU** |
| --- | --- |
| Root – associated soil | ~3.48×10^8^ |
| Root Surface | ~1.77×10^8^ |
| Root tissues | ~3.6×10^7^ |
